# A model for dinitrogen binding in the E_4_ state of nitrogenase†
†Electronic supplementary information (ESI) available: Further information on all broken-symmetry solutions calculated for all models. Details of the QM/MM model preparation. Spin populations of all models. Localized orbital analysis of selected models. Geometry analysis of E_0_ state calculated with different functionals and electronic structure analysis. Cartesian coordinates for the QM regions of all optimized structures available as XYZ files. See DOI: 10.1039/c9sc03610e


**DOI:** 10.1039/c9sc03610e

**Published:** 2019-10-15

**Authors:** Albert Th. Thorhallsson, Bardi Benediktsson, Ragnar Bjornsson

**Affiliations:** a Science Institute , University of Iceland , Dunhagi 3 , 107 Reykjavik , Iceland; b Department of Inorganic Spectroscopy , Max-Planck-Institut für Chemische Energiekonversion , Stiftstrasse 34-36 , 45470 Mülheim an der Ruhr , Germany . Email: ragnar.bjornsson@cec.mpg.de

## Abstract

Molybdenum nitrogenase is one of the most intriguing metalloenzymes in nature, featuring an exotic iron–molybdenum–sulfur cofactor, FeMoco, whose mode of action remains elusive. In particular, the molecular and electronic structure of the N_2_-binding E_4_ state is not known. In this study we present theoretical QM/MM calculations of new structural models of the E_4_ state of molybdenum-dependent nitrogenase and compare to previously suggested models for this enigmatic redox state. We propose two models as possible candidates for the E_4_ state. Both models feature two hydrides on the FeMo cofactor, bridging atoms Fe_2_ and Fe_6_ with a terminal sulfhydryl group on either Fe_2_ or Fe_6_ (derived from the S2B bridge) and the change in coordination results in local lower-spin electronic structure at Fe_2_ and Fe_6_. These structures appear consistent with the bridging hydride proposal put forward from ENDOR studies and are calculated to be lower in energy than other proposed models for E_4_ at the TPSSh-QM/MM level of theory. We critically analyze the DFT method dependency in calculations of FeMoco that has resulted in strikingly different proposals for this state. Importantly, dinitrogen binds exothermically to either Fe_2_ or Fe_6_ in our models, contrary to others, an effect rationalized *via* the unique ligand field (from the hydrides) at the Fe with an empty coordination site. A low-spin Fe site is proposed as being important to N_2_ binding. Furthermore, the geometries of these states suggest a feasible reductive elimination step that could follow, as experiments indicate. *Via* this step, two electrons are released, reducing the cofactor to yield a distorted 4-coordinate Fe_2_ or Fe_6_ that partially activates N_2_. We speculate that stabilization of an N_2_-bound Fe(i) at Fe_6_ (not found for Fe_2_ model) *via* reductive elimination is a crucial part of N_2_ activation in nitrogenases, possibly aided by the apical heterometal ion (Mo or V). By using protons from the sulfhydryl group (to regenerate the sulfide bridge between Fe_2_ and Fe_6_) and the nearby homocitrate hydroxy group, we calculate a plausible route to yield a diazene intermediate. This is found to be more favorable with the Fe_6_-bound model than the Fe_2_-bound model; however, this protonation is uphill in energy, suggesting protonation of N_2_ might occur later in the catalytic cycle or *via* another mechanism.

## Abstract 2

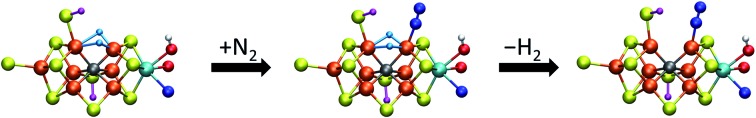
QM/MM calculations are used to propose a new model for the E_4_ state of FeMoco and how N_2_ binding to this state may occur.

## Introduction

**Fig. 1:**
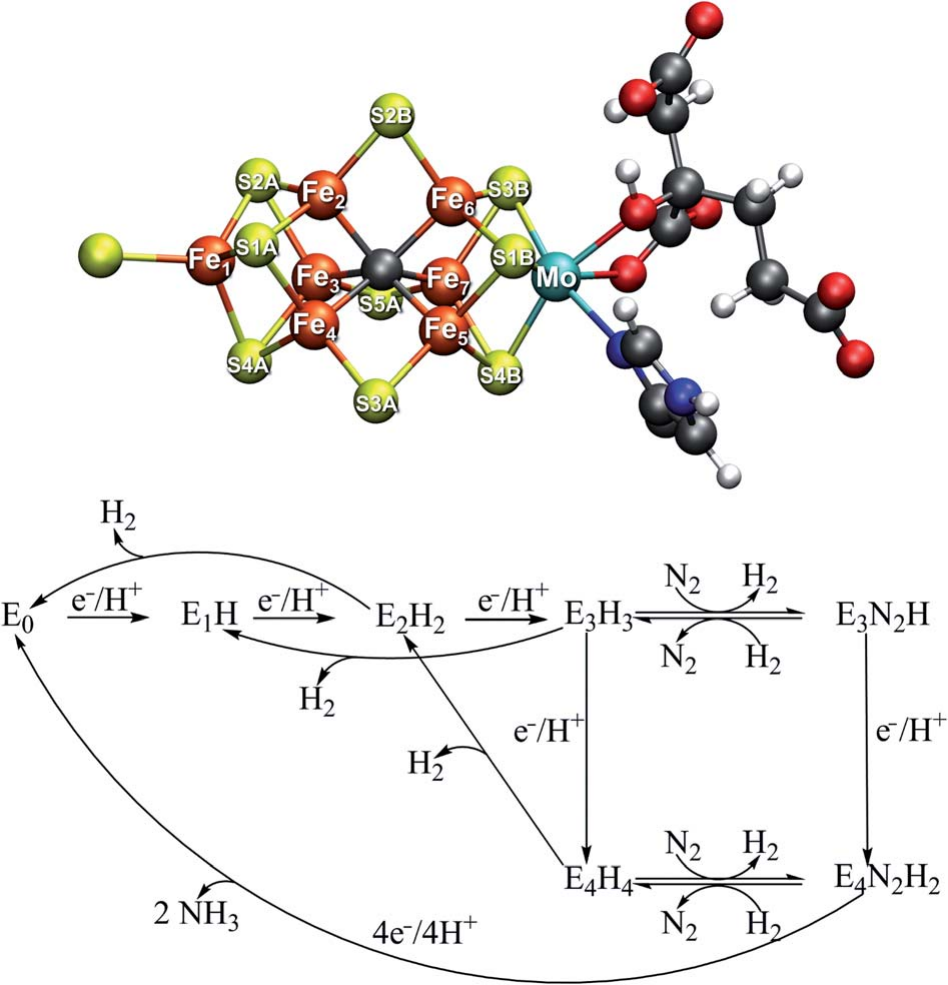
Top: Resting state structure of the iron–molybdenum cofactor (FeMoco) in the active site of MoFe protein of Mo-dependent nitrogenase. The cofactor is bound to the α-275^Cys^ residue at Fe_1_ and the α-442^His^ residue at Mo. Bottom: A simplified Lowe–Thorneley scheme showing the early redox states of FeMoco up until dinitrogen binding. While H_2_ evolution can occur as side-reactions from the E_2_, E_3_ and E_4_ states, obligatory H_2_ evolution is proposed to occur concomitantly as N_2_ binds in the E_4_ state.

The mechanism of biological nitrogen reduction, catalyzed by the bacterial nitrogenase enzymes, has remained an unsolved problem in bioinorganic chemistry despite decades of research involving many experimental and theoretical research groups. The nitrogenases are metalloenzymes that catalyze the reduction of dinitrogen to two molecules of ammonia for each molecule of dinitrogen, impressively at ambient conditions.^1^,^2^ The molybdenum-dependent form is the most active and the best characterized, yet there is no consensus on the dinitrogen binding site on the cofactor nor is there a basic mechanism in place. The complete enzyme consists of a transient complex of the MoFe protein and the Fe protein. The function of the Fe protein is electron transfer to the MoFe protein *via* an [Fe_4_S_4_] cluster in an ATP-dependent process while dinitrogen reduction takes place in the MoFe protein. The active site of the MoFe protein, includes a complex [MoFe_7_S_9_C] cofactor (FeMoco, shown in Fig. 1) where dinitrogen binds. The kinetic studies by Lowe and Thorneley^3^ resulted in a model for dinitrogen catalysis linking different redox states of the cofactor (denoted E_*n*_, with *n* indicating the number of added electrons and protons) together. The obligatory formation of one molecule of H_2_ per molecule of N_2_ is an intriguing aspect of the model, suggesting the unusual stoichiometry and the apparent waste of 2 electrons and 4 molecules of MgATP to produce this extra molecule of H_2_.^4^N_2_ + 8e^–^ + 8H^+^ + 16MgATP → 2NH_3_ + H_2_ + 16MgADP

Curiously, in the Lowe–Thorneley model (a simplified scheme is shown in Fig. 1), dinitrogen does not bind to the iron–molybdenum cofactor until either the E_3_ or E_4_ state (after 3 or 4 added electrons and protons to the resting state E_0_), concomitant with formation of one molecule of H_2_. This obligatory H_2_ evolution step (as N_2_ binds) is thus directly part of the mechanism (rather than being one of the known side-reactions).^4^ Most kinetic and spectroscopic studies have been directed towards the E_4_ state as it is EPR active, its population can be increased by specific mutations and it has been argued to be the primary state for N_2_ binding.^3^,^4^

The FeMoco cluster is a highly unusual metallocofactor with a molecular structure that took many years to characterize; it consists of 7 iron ions, 1 molybdenum ion, 9 sulfides and an interstitial carbide^5^,^6^ (see Fig. 1). Its electronic structure is deeply complicated owing to the open-shell nature of the metal ions and their complex spin coupling in a highly covalent cluster (including an unusual carbide ligand).^7^ The iron oxidation states of the resting state FeMoco are formally Fe(ii) and Fe(iii) (typical in iron–sulfur clusters) while the Mo ion was determined *via* Mo K-edge and L-edge XAS spectroscopy to be in a Mo(iii) oxidation state and theoretical calculations have shown it to be in an unusual spin-coupled low-spin state, likely due to metal–metal bonding interactions with the Fe ions.^8^,^9^ Recent X-ray magnetic circular dichroism experiments demonstrated the presence of the unusual spin-coupled state of Mo(iii) in a related [MoFe_3_S_4_] model cubane.^10^ The charge of the cofactor in the resting state has been determined to be [MoFe_7_S_9_C]^1–^ according to Mössbauer analysis, spatially resolved anomalous dispersion refinement and comparison of calculated and crystallographic metal–metal distances.^11^–^13^ Recent studies of the electronic structure from broken-symmetry (BS) DFT studies reveals a complicated electronic structure featuring both antiferromagnetic coupling, mixed-valence delocalization and partial metal–metal bonding.^8^,^11^,^13^

Other redox states of FeMoco are less well-characterized but we note recent spectroscopic studies of the E_1_ state^14^,^15^ and the E_2_ state.^16^,^17^ The binding site of dinitrogen is far from obvious from inspection of the cluster in its resting state (E_0_) and since little is known about the structure of the E_4_ redox state (which has been argued to be the primary state for N_2_ binding during turnover^3^,^4^), this is one of the most pressing questions in nitrogenase research. Even less is known about the E_3_ state and its proposed N_2_ binding as the state is EPR silent. A correct structural model for E_4_ would arguably reveal (or at least strongly suggest) how dinitrogen can favourably bind to FeMoco and how this typically inert molecule is activated for protonation. An accumulated *S* = 1/2 E_4_ state was originally probed by EPR spectroscopy in a mutant (α-70^Val→Ile^) of MoFe protein and ENDOR spectroscopy revealed that the structure contains 2 chemically near-equivalent bridging hydrides, likely storing the 4 reducing equivalents.^18^ Later, the EPR signal and ENDOR hyperfine signals associated with this state were found in the wild-type protein as well.^19^ The reductive elimination of H_2_ from these two hydrides was furthermore proposed to explain the obligatory H_2_ evolution in the mechanism^4^,^20^ and how dinitrogen is activated for protonation and there is now ample experimental evidence for this proposal.^19^,^21^ There is, however, no consensus on the structure of the E_4_ state, neither the precise coordination of the hydrides, nor is the N_2_ binding site agreed upon. Mutation studies have previously implicated the Fe_2_–Fe_3_–Fe_6_–Fe_7_ face as a likely binding site for dinitrogen as no dinitrogen reduction is observed when the residue α-70^Val^ is mutated into the bulkier α-70^Ile^.^22^ Recent joint experimental–computational studies have proposed E_4_ models featuring bridging hydrides^23^–^25^ (the favoured model features bridging hydrides between Fe_3_–Fe_7_ and Fe_2_–Fe_6_) while other computational studies have suggested mixed bridging/terminal hydride models^26^,^27^ or even, surprisingly, structures featuring a protonated carbide with/without bridging hydrides.^28^–^31^ Additionally, recent crystal structures of a CO-bound form^32^ of MoFe protein (CO being an inhibitor) and an XH-ligand-bound (X = N or O) form^33^ of VFe protein have emerged, that show CO/XH replacing the Fe_2_–Fe_6_ bridging sulfide in a bridging binding mode (we recently showed *via* QM/MM calculations that the XH ligand in the FeVco structure is more consistent with an OH).^34^ These crystal structures demonstrate the lability of the sulfide bridges, which hints at a possible binding site of dinitrogen or perhaps a binding site of hydrides as discussed by Einsle *et al.*^33^,^35^ Overall, there is no consensus on the nature of the E_4_ state.

The present study proposes new structural models for the E_4_ state of FeMoco as well as a model for dinitrogen binding based on theoretical calculations. A state-of-the-art QM/MM protocol using the broken-symmetry DFT approach is used that has been previously validated on the resting E_0_ state.^13^ We compare the new structural models to previous proposals by calculating all models at the same QM/MM level of theory. The DFT method dependence of the relative energies is discussed and is put into context with the ability of different computational protocols to describe the electronic structure of the resting state correctly. Two energetically favourable models appear to be consistent with the bridging hydride proposal from experimental ENDOR studies and while favourable dinitrogen binding to both is possible, the E_4_ model featuring an open coordination site at Fe_6_ binds N_2_ slightly stronger. Concomitant H_2_ evolution with N_2_ binding *via* the reductive elimination proposal can be explained using our model due to close proximity of the hydrides (favoured over a hydride-proton reaction) and this leads to the stabilization of an N_2_-bound Fe(i) intermediate at Fe_6_ (or alternatively to an N_2_-bound Fe(ii) intermediate at Fe_2_). A possible protonation step to yield a FeMoco-bound diazene intermediate is discussed.

## Computational details

The QM/MM models for E_4_ were based on our model for the E_0_ resting state model that has been previously described.^13^ It is a spherical QM/MM model (42 Å radius and ∼37 000 atoms) centered on the carbide of FeMoco that includes roughly half of the tetrameric MoFe protein; see ESI† for details about the QM/MM model preparation. In the QM/MM geometry optimizations of E_4_ models the active region consists of 1003 atoms and a QM region of 136 atoms while calculations of E_0_ used a smaller QM region of 54 atoms. All QM/MM calculations were performed in Chemshell^36^,^37^ using the built-in MM code DL_POLY^38^ with the CHARMM36 forcefield^39^ and ORCA version 4.0 (ref. 40) as QM code. The 136 atom QM region (see Fig. S4 in ESI†) contains the FeMoco cofactor, singly protonated homocitrate and the sidechains of residues α-70^Val^, α-96^Arg^, α-191^Gln^, α-195^His^, α-275^Cys^, α-278^Ser^, α-359^Arg^, α-380^Glu^, α-381^Phe^ and α-442^His^. The QM region thus contains the main residues critical to describing the coordination, electrostatic environment, asymmetry, and hydrogen-bonding environment around FeMoco. This includes residues directly coordinating FeMoco (α-442^His^, α-275^Cys^), neighboring charged residues (α-96^Arg^, α-359^Arg^, α-380^Glu^), those capable of participating in hydrogen bonding (α-195^His^, α-191^Gln^, α-278^Ser^), as well as spatially close residues (α-70^Val^, α-381^Phe^). α-195^His^ is calculated to be in the N_δ_ protonation state, assuming the residue has donated a proton from N_ε_ to the cofactor and has been reprotonated at N_δ_. All QM/MM calculations used electrostatic embedding, and hydrogen link atoms were used to terminate the QM–MM border together with the charge-shift procedure as implemented in Chemshell. The E_4_ QM calculations primarily used the TPSSh hybrid density functional,^41^,^42^ a ZORA scalar relativistic Hamiltonian,^43^,^44^ the relativistically recontracted def2-TZVP basis set^45^,^46^ on all metal, sulfur, carbide, homocitrate and hydride/SH/CH atoms (def2-SVP on other atoms) and a D3BJ dispersion correction.^47^,^48^ The RIJCOSX approximation^49^,^50^ with a decontracted Coulomb auxiliary basis set by Weigend^51^ was used. All DFT calculations used tight integration grids (Grid5 Finalgrid6 settings in ORCA). Single-point energy calculations of E_4_ models were performed at the TPSS,^41^ B3LYP^52^–^55^ and M06-2X^56^ levels using the same basis set. M06-2X calculations used a very large grid (Grid7 in ORCA) due to the grid-dependence associated with this functional.^57^ QM/MM geometry optimizations of the E_0_ state with other functionals always used the D3BJ dispersion correction except in the case of M06-2X where a D3(0) correction was used. Functionals used for E_0_ geometry optimizations were BP86,^52^,^58^ TPSS,^41^ B3LYP, PBE0,^59^,^60^ BHLYP,^61^ M06-2X and ωB97M-D3BJ.^62^,^63^ Broken-symmetry solutions of the E_4_ models were found by flipping spins on Fe atoms converging to the *M*_S_ = 1/2 solution. Four different broken-symmetry solutions were explored (with Fe ions 235, 346, 247 or 147 spin down; atom numbering as in crystal structure) as discussed later. Vibrational frequencies of N_2_-bound models were calculated from a numerical QM/MM partial Hessian. While all QM/MM calculations minimized the QM/MM energy function, we primarily discuss the polarized QM energy rather than the total QM/MM energy as the former is less sensitive to the unrelated MM energy changes of the MM region.

## Results and discussion

### A. Computational modelling of the E_4_ state

Theoretical modelling of the E_4_ state (here defined as the state after addition of 4e^–^ and 4H^+^ to the resting state E_0_ of FeMoco) has been pursued recently by many groups and their models will be discussed and compared. It is helpful to first discuss criteria that, in our view, a realistic model for the E_4_ state should ultimately fulfil. These criteria are: (i) consistency with available spectroscopic data, (ii) the model being a thermodynamically viable state, (iii) demonstration of favorable N_2_ binding and consistency with isotope substitution experiments and, (iv) computational consistency.

Regarding criterion i, regrettably, spectroscopic data for the E_4_ state are scarce and are primarily available in the form of EPR and ENDOR data, and primarily on a mutant form of the protein (which appears though to have the same spectroscopic signature as the wild type^19^). The EPR data indicates a spin state of *S* = 1/2 and analysis of the ^1^H hyperfine tensors from ^1^H ENDOR spectroscopy led to the proposal that the state contains two near-identical metal-bound hydrides, that are bridging rather than terminal and bound to Fe ions rather than the Mo ion according to ^95^Mo ENDOR.^64^ Furthermore, ^57^Fe ENDOR indicates that the overall iron redox level of E_4_ is the same as in the E_0_ state,^65^ which suggests that the two hydrides are acting as carriers of the four added electrons. A recent high-resolution ENDOR study of the same state has additionally revealed signals from two other hydrogens, likely present as protons bound to sulfides.^25^ As quantum chemical calculations of FeMoco are currently mostly limited to broken-symmetry DFT approaches (where pure spin states are not calculated) and spin projection schemes are problematic for non-Heisenberg systems like FeMoco, this unfortunately makes a direct comparison of the calculations to magnetic spectroscopy data from EPR and ENDOR difficult. However, an indirect comparison is still possible, *i.e.* a comparison of computed structures to the bridging hydride structural motif suggested by the ENDOR analysis.

Regarding thermodynamic stability *i.e.* criterion ii, kinetic studies of MoFe protein under turnover, indicates the E_4_ state is only fleetingly stable. The state can be freeze-trapped in a turnover sample with a small population but will evolve H_2_ with fast rates to fall back to an E_2_ state (that in turn further evolves H_2_ and falls back to E_0_).^66^ The fleeting nature of this state and the fact that the mechanisms of reduction and protonation are not entirely clear, indicates that thermodynamics alone cannot be the only guiding principle for differentiating between models for E_4_; however, thermodynamic stability must still be relevant to its formation.

Criterion iii implies that the model should ideally demonstrate favourable N_2_ binding. Experimental studies indicate N_2_ binding to be unfavourable in the early redox states, suggesting that the unknown E_4_ state features a structural component that makes N_2_ binding favourable (*e.g.* an empty coordination site), unlike redox states E_0_–E_2_ (E_3_ has also been proposed to bind N_2_). Furthermore, a model for E_4_ should offer a plausible explanation or mechanism for the obligatory H_2_ evolution resulting from the reductive elimination of H_2_ from two hydrides (as shown by isotope substitution studies) as N_2_ binds to FeMoco.

Finally, we propose computational consistency as criterion iv, that the computational E_4_ model should satisfy. By this, we mean that the computational protocol used to propose the model should also be shown to be consistent with other important experimental data of the system such as data for the other redox states. The 1.0 Å crystal structure of the E_0_ state^6^ is in our view the most accessible experimental data for gauging the quality of the computational protocols. The quality of the crystal structure of MoFe protein has improved steadily in recent years and has a bond length uncertainty of ∼0.02 Å. We consider a satisfactory agreement of the computed resting state structure to the crystal structure to be vital to any computational protocol that is used to suggest new redox state models of FeMoco.

All models that we consider in this study were structurally optimized at the same QM/MM level of theory that we have previously used to describe the resting state E_0_ and we considered multiple broken-symmetry states for each model. As recently discussed by Raugei *et al.*,^24^ the protein environment and the quality of the computational model can make a surprisingly large difference regarding the relative stability of an E_4_ isomer. Raugei *et al.* demonstrated *e.g.* that a protonated-carbide model for E_4_ was only stable for small cluster models of the active site but became unstable when larger cluster models were considered. As our QM/MM model accounts for the explicit protein environment from the beginning (and avoids potentially artificial constraints on residues) and furthermore utilizes a large QM-region (including important charged and hydrogen-bonding residues near FeMoco), our calculations should suffer much less from such artifacts. Our QM/MM methodology has previously been validated by detailed comparison of the calculated molecular structure of the cofactor in the resting state to the available high-resolution crystal structure, giving better agreement for the basic metal framework than cluster models.^13^ As before, we primarily utilize the TPSSh level of theory (a 10% Hartree–Fock (HF) exchange TPSS hybrid), a ZORA scalar relativistic Hamiltonian, dispersion correction and a flexible polarized triple-zeta basis set (on the cofactor). The functional dependency of calculations is discussed in chapter C.

**Fig. 2:**
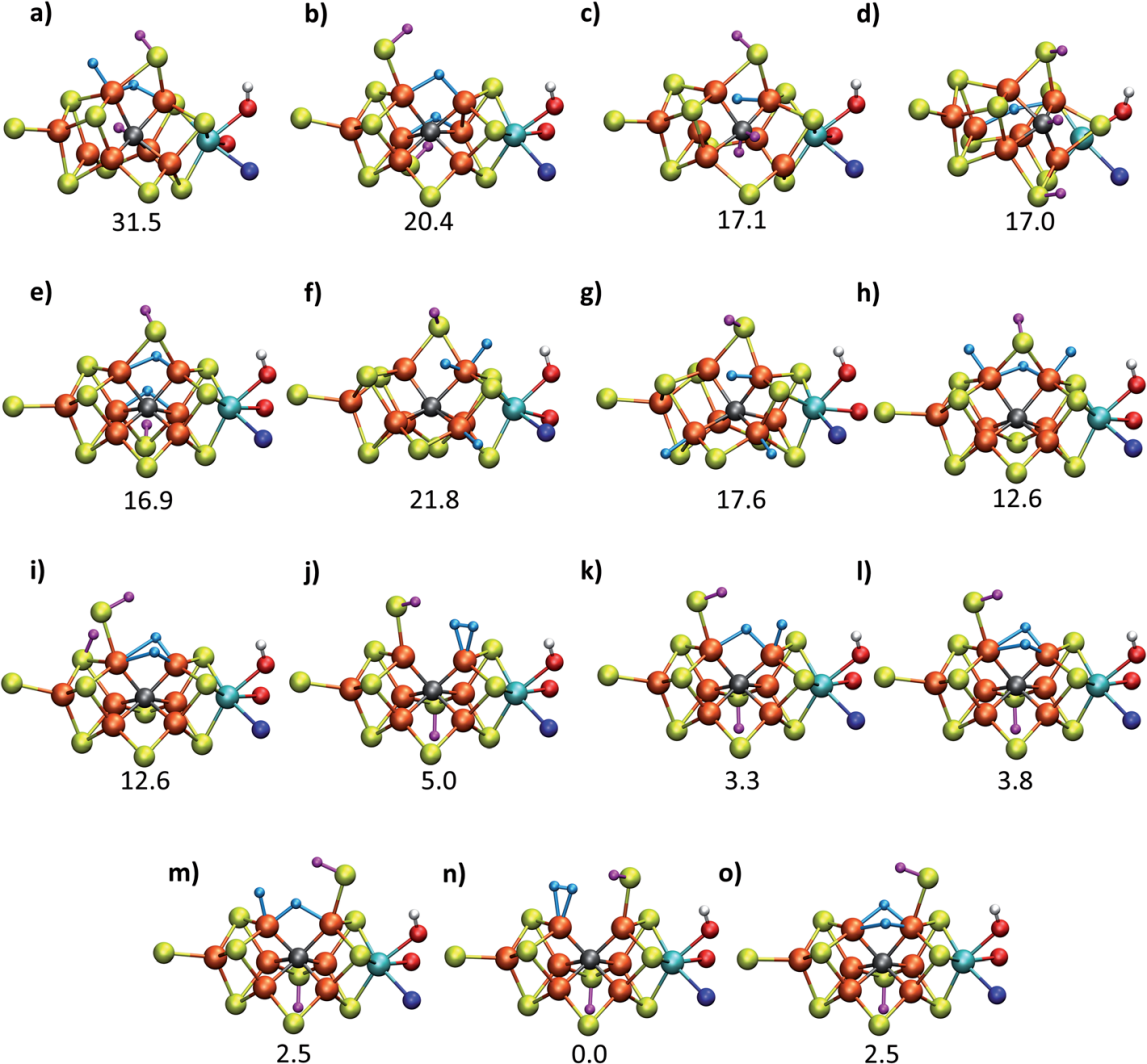
Structures and relative polarized electronic energies (*E*_QM_ in kcal mol^–1^) of all FeMoco models for the E_4_ state considered in this study. All models were minimized using the same QM/MM level of theory using the TPSSh-D3 functional, ZORA–def2-TZVP basis set and a ZORA scalar relativistic Hamiltonian. See Tables 1 and S1 and S2† for information on all BS solutions tested. A large QM region of 136 atoms was used in the calculations but only the cofactor geometry is shown here. Hydrides are colored in light blue and carbide/sulfide-bound protons in magenta.

Due to the complex open-shell nature of the [MoFe_7_S_9_C] cluster, the spin coupling problem of FeMoco is far from trivial. The broken-symmetry DFT methodology used here, flips individual spins on atoms and converges to broken-symmetry SCF solutions with localized alpha and beta spins on different atoms. These BS states likely correspond well to some of the low energy electronic states of FeMoco, but the methodology cannot capture the full multiplet spectrum of such a complex spin-coupled metal-sulfur cluster. Additionally, as discussed in recent multiconfigurational wavefunction theory studies, simplified model Hamiltonians (Heisenberg and Double Exchange Hamiltonians) are likely too simple to give a realistic description for spin-coupled iron–sulfur systems,^67^ preventing the use of spin-projection schemes. For the resting state, the BS7 class of solutions (that appears to maximize antiferromagnetic interactions) has been consistently found to be most favourable in multiple studies^5^,^8^,^68^–^71^. The three BS7 solutions, here labelled according to which Fe ions are spin-down (crystal structure numbering): BS-235, BS-346 and BS-247 have been found to be within ∼1 kcal mol^–1^ of each other (QM/MM-TPSSh level of theory) and in previous work^13^ we found that the BS-235 solution was in better agreement with the crystal structure than the other spin isomers. As E_4_ is a different redox state, however, the spin coupling may well have changed completely due to binding of hydrides and in fact the experimental spin state changes from *S* = 3/2 (E_0_) to *S* = 1/2 (E_4_). Thus a similar BS state to the one in E_0_ cannot simply be assumed to apply to E_4_. Cao *et al.*^27^ have shown, however, in their work where many different E_1_–E_4_ models were considered (featuring many of the models studied herein) that the BS solutions that are consistently lowest in energy are the BS7 class of solutions, thus supporting the hypothesis that maximizing antiferromagnetic coupling remains important, even when hydrides are bound. In this work we have primarily considered the BS7 solutions (BS-235, BS-346 and BS-247) as the most relevant broken-symmetry solutions as well as the BS-147 solution. The BS7 solutions are similar in energy and feature a very similar electronic structure in the resting state E_0_ (as discussed in ref. 11 and 13); the differing positions of spin-down Fe atoms though has the effect of changing the iron oxidation state at each site, as the mixed-valence pairs or localized ferric/ferrous irons will then involve different atoms in the structure. This local oxidation state interpretation of the different broken-symmetry states has been previously discussed.^11^,^13^,^15^ A BS-147 solution features ferromagnetic alignment of atoms Fe_2_ and Fe_6_. As some of the models in this study feature structural changes at these Fe ions, it seems possible that such a solution could become more favourable than other BS solutions. To aid in the computational problem involving many models and many BS states at an expensive level of theory, we have restricted ourselves to performing geometry optimizations with the BS-235, BS-247, BS-346, BS-147 solutions to explore the energetics of all models in Fig. 2. We additionally explored all 35 BS solutions of two models (**l** and **o**) at the single-point level (see ESI, Fig. S5 and S6† respectively). All models assumed a final *M*_S_ = 1/2 spin state, consistent with the experimental *S* = 1/2 spin state of E_4_.

### B. QM/MM calculations of models of the E_4_ state

Fig. 2 shows the molecular structures of various proposed E_4_ models from previous work and new ones, featuring a variety of hydride binding modes and/or sulfide/carbide protonation scenarios. We are aided here by the work of Cao *et al.*,^27^ who performed a systematic study of a large number of protonation positions in the E_0_–E_4_ redox states. Thus, we have included the most stable E_4_ models found in their study (the study lacks, however, open sulfide-bridge models) according to the levels of theory employed in their study (TPSS and B3LYP), as well as models from other groups. All models, shown in Fig. 2, can be grouped according to whether they contain a protonated carbide (model **a**, **c** and **d**),^27^,^29^,^30^ whether they feature terminal/bridging hydrides with an intact sulfide bridge (models **e**, **f**, **g** and **h**)^24^,^26^,^27^ or whether they feature hydrides with an open sulfide bridge in the form of a terminal sulfhydryl group (models **b**, **i**, **j**, **k**, **l**, **m**, **n**, **o**).

Models **n**, **m** and **o** were previously discussed as models close in energy to the favoured model **e** in work by Raugei *et al.* In this study we introduce models **l** and model **o** (though a model like **o** has been discussed by Raugei *et al.*^24^) as plausible candidates for E_4_ as well as models **j** and **k**, being similar to models **m** and **n**, and model **b** featuring two terminal sulfhydryl groups and two bridging hydrides. Model **i** also has a terminal sulfhydryl group like **l** but has a different sulfide protonation state.^72^ The models with a terminal sulfhydryl group at Fe_2_/Fe_6_ (rather than at Fe_4_/Fe_5_ or Fe_3_/Fe_7_) seem consistent with crystal structures of ligand-bound states^32^,^33^ lacking S2B (that bridges Fe_2_ and Fe_6_), suggesting some lability of this particular sulfide bridge. Fig. 2 and Table 1 contains data for the lowest energy BS solution but data for all broken-symmetry solutions can be found in Tables S1–S5† along with Mulliken spin populations for each state.

**Table 1:** Calculated relative energies (kcal mol^–1^) of the E_4_ models investigated (shown in Fig. 2) with different functionals, with the lowest energy broken symmetry solution indicated for each model. For TPSSh, both the polarized QM energies (MM point charges included in the QM calculation) and total QM/MM energies are given. Single-point (polarized) QM calculations with TPSS, B3LYP and M06-2X used the TPSSh geometries. See Tables S2–S5 in the ESI for data on all BS solutions calculated

TPSSh			Single-point QM energies (*E*_QM_)					
Model (BS-state)	*E*_QM_	*E*_QM/MM_	Model	TPSS	Model	B3LYP	Model	M06-2X
**a** (147)	31.46	23.67	**a** (235)	37.77	**a** (147)	36.83	**a** (147)	59.26
**b** (247)	20.40	17.66	**b** (247)	15.77	**b** (147)	26.84	**b** (346)	47.33
**c** (247)	17.14	14.73	**c** (247)	36.81	**c** (147)	2.46	**c** (147)	0.00
**d** (147)	16.96	17.58	**d** (147)	26.12	**d** (346)	15.70	**d** (147)	41.68
**e** (147)	16.88	11.23	**e** (147)	12.81	**e** (235)	34.70	**e** (235)	61.35
**f** (346)	21.80	22.55	**f** (346)	3.43	**f** (235)	43.19	**f** (235)	114.39
**g** (346)	17.60	13.02	**g** (346)	4.07	**g** (235)	38.21	**g** (147)	104.64
**h** (247)	12.56	11.37	**h** (346)	2.87	**h** (247)	33.04	**h** (247)	100.05
**i** (247)	12.55	10.91	**i** (247)	12.08	**i** (247)	19.60	**i** (247)	58.24
**j** (147)	4.99	3.35	**j** (235)	3.50	**j** (147)	12.34	**j** (247)	31.41
**k** (147)	3.29	1.95	**k** (235)	0.37	**k** (147)	13.18	**k** (147)	57.42
**l** (147)	3.75	2.44	**l** (235)	2.28	**l** (147)	14.52	**l** (147)	55.05
**m** (346)	2.47	2.04	**m** (235)	0.21	**m** (346)	0.00	**m** (346)	10.29
**n** (346)	0.00	0.00	**n** (346)	6.73	**n** (346)	1.40	**n** (235)	26.54
**o** (147)	2.48	2.25	**o** (147)	0.00	**o** (346)	12.96	**o** (346)	44.50

As shown in Fig. 2 and Table 1, the relative energies (polarized QM energies at QM/MM geometries) at the TPSSh level of theory indicate models featuring protonated carbides (**a**, **c**, **d**) are strongly disfavoured, appearing much too high in energy (17–32 kcal mol^–1^ higher in energy than the lowest energy model **n**) to be likely candidates for the E_4_ state. Additionally, models **e**, **f**, **g**, **h**, featuring an intact sulfide bridge also appear rather high in energy ranging from 13–22 kcal mol^–1^. Only open sulfide bridge models, **j–o**, featuring a terminal sulfhydryl group at Fe_2_ or Fe_6_ are found to be similarly low in energy, suggesting that it is generally thermodynamically favourable to alter the coordination of a protonated sulfide bridge to aid stabilizing hydrides (bridging, terminal or dihydrogen-like). Model **b** with two open sulfide bridges is an exception to this trend. This energetic analysis suggests that models **j–o** are the most viable models for the E_4_ state (at the TPSSh-QM/MM level). Open sulfide-bridge models like **j–o** were not considered in the study by Cao *et al.*^27^ but some open-sulfide bridge models were discussed by Raugei *et al.*^24^ and were found to be similar in energy as model **e**, which, however, is not in agreement with our results. We attribute this disagreement to different modelling aspects, *i.e.* cluster modelling *vs.* QM/MM modelling, as well as BS-solution dependence and functional dependence, which will be discussed in the next section.

### C. Computational protocol dependence

As has recently been discussed in the literature, by Raugei *et al.*^24^ and especially by Cao *et al.*^27^,^73^ there is a considerable functional dependency present in calculations of FeMoco, affecting *e.g.* whether carbide protonation is favoured or not, or the structure of FeMoco. Thus, when hybrid density functionals with ≥20% HF exchange such as B3LYP (20% HF exchange) and M06-2X (54% HF exchange) have been used in the computational protocol, carbide protonation models have been found to be favoured over models with only hydrides. This is at odds with our relative energy comparison at the TPSSh level (10% HF exchange), shown in Fig. 2. We have been able to confirm some of this behaviour by performing single-point energy calculations using these functionals with the TPSSh-optimized geometries. As shown in Table 1, the energy comparison of models is dramatically altered when these hybrid functionals are considered. Meanwhile, results from the non-hybrid functional TPSS are more similar to the TPSSh results. As an example of this strong functional dependency, model **c** (a protonated carbide model found in the study by Cao *et al.*), is predicted to be only ∼2.5 kcal mol^–1^ higher in energy than the lowest energy model (**m**) at the B3LYP level of theory but ∼17 kcal mol^–1^ higher in energy than the lowest model (**n**) at the TPSSh level of theory. Structural optimization at the same level of theory would likely further stabilize model **c**. This effect of using a higher HF exchange hybrid is further magnified when using the M06-2X functional (having 54% HF exchange); this even leads to model **c** becoming the most stable model as seen in Table 1 (and the energy gap between models becoming 114 kcal mol^–1^). The M06-2X functional has been used to suggest carbide protonation as an important aspect of the mechanism.^29^

**Fig. 3:**
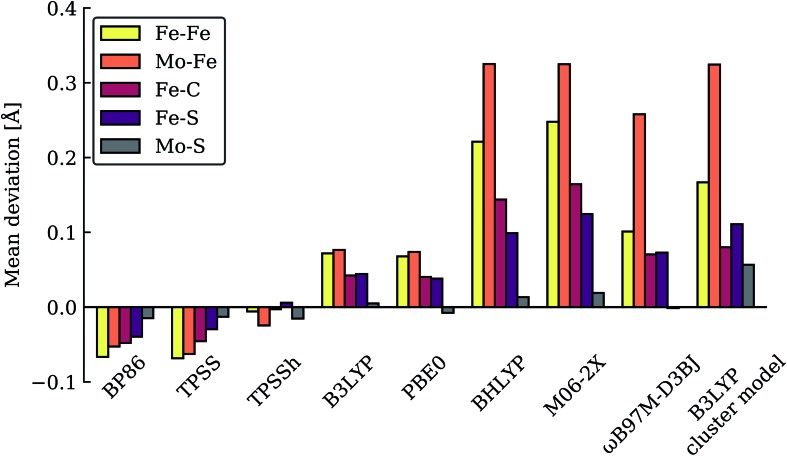
Mean deviations (Å) of Fe–Fe, Mo–Fe, Fe–C, Fe–S and Mo–S distances of resting state FeMoco (relative to crystal structure), calculated with various functionals using the same QM/MM protocol. Also shown is a B3LYP cluster model from Siegbahn.^74^

The large energy changes seen for different functionals echo the results of Cao *et al.*^73^ We also note the pronounced sensitivity w.r.t. which BS-solution is calculated (see Tables S1–S2†), underlining the importance of studying multiple BS states in calculations of FeMoco. Such a large functional dependency of the relative energies (even when the same TPSSh geometries are used) naturally calls into question the reliability of the DFT calculations to distinguish energetically between E_4_ models of these complex systems. In fact, these results strongly suggest that the different functionals are describing the electronic structure of the FeMoco E_4_ state very differently. Such a different description of electronic structure with different functionals for FeMoco in the E_4_ state should be apparent in the E_0_ state as well (despite an absence of hydrides and protonated sulfides/carbide). In fact, when different functionals are used to describe the electronic structure of FeMoco in the E_0_ state, there are notable differences in the spin populations and isosurface plots of the spin density (see ESI, Fig. S13 and S14, Tables S9 and S11†). These differences can partly be attributed to the differing delocalization of electrons in FeMoco as well as the strength of Mo–Fe interactions (as discussed in previous studies the Mo electrons are partially delocalized towards the Fe ions, suggesting some Mo–Fe bonding^7^,^8^,^11^,^13^). A functional such as B3LYP for example shows a slightly more localized description of unpaired electrons in FeMoco and this effect is magnified when going to functionals with more HF exchange like BHLYP or M06-2X. In fact, when QM/MM geometry optimizations of the E_0_ state are performed with hybrid functionals with HF exchange ≥20% (such as B3LYP, BHLYP and M06-2X), large structural changes occur. This can be seen for Mo–Fe, Fe–Fe and Fe–C distances in Fig. 3 which shows the mean deviation of specific atom–atom distances w.r.t. crystal structure (more data available in the ESI, Fig. S10–S12†). Especially noteworthy is the up to 0.8 Å deviation seen for the Mo–Fe_1_ distance (see ESI, Fig. S10†) that suggests that the basic structure of the cofactor is very badly described at some of these same hybrid levels. As discussed further in the ESI,† the largest structural deviations involve Mo–Fe distances, and for BHLYP and M06-2X, this can be attributed to a completely different electronic structure on the molybdenum, giving rise to a high-spin Mo(iv) instead of the non-Hund Mo(iii) configuration. The oxidation state of Mo as Mo(iii) in FeMoco is now firmly established from Mo K-edge and L-edge XAS spectroscopy and theoretical calculations.^8^,^9^ Additionally, the unusual spin-coupled non-Hund Mo(iii) configuration, first proposed by theoretical calculations^8^ has now experimental support *via* recent Mo L-edge and XMCD spectra.^10^ Thus, computational protocols giving rise to long Mo–Fe_5_/Fe_6_/Fe_7_ distances (>3 Å) by stabilizing a high-spin Mo(iv) ion are not only incompatible with the experimental molecular structure data (high resolution crystallography) but experimental electronic structure data (XAS and XMCD) as well. The QM/MM B3LYP results in Fig. 3 and the ESI† show considerable deviations with respect to the crystal structure (though not as large as the BHLYP and M06-2X results); however, this effect is further magnified if the QM/MM model is replaced by a simpler cluster model instead. The B3LYP cluster model data shown in the figures are from Siegbahn^74^ and give deviations as bad as the BHLYP and M06-2X QM/MM data. As shown in the ESI† there are also dramatic changes in the electronic structure of the B3LYP cluster model, especially regarding the oxidation state of Mo, again indicating a Mo(iv) oxidation state. It is also worth noting that overestimated Fe–C bond lengths (suggesting destabilized Fe–C chemical bonds) are seen from functionals/protocols that show more favourable carbide protonation according to Table 1 and these functionals/protocols have been used in studies that suggest carbide protonation occurring in reduced states of FeMoco. The non-hybrid functionals, BP86 and TPSS, give structures in better agreement with the experimental crystal structure (though showing underestimation of distances instead of overestimation). TPSS shows relative energies of E_4_ models more similar to TPSSh than the hybrid functionals, at least regarding the stability of carbide-protonation models. FeMoco is thus an interesting example of where there is quite a strong relationship between electronic structure (and hence reaction energies) and molecular structure.

The good agreement seen between our TPSSh-QM/MM E_0_ structure and the high resolution crystal structure of E_0_ strongly suggests that we are describing the electronic structure of the system more correctly with TPSSh, clearly much better than the higher exchange hybrids and also better than non-hybrid functionals such as BP86 and TPSS. We note that our calculations include dispersion corrections, a large polarized triple-zeta basis set (on the cofactor) that should be close to the basis set limit, as well as scalar relativistic effects (*via* ZORA) and we account for the explicit protein environment from the beginning; the good agreement seen with our TPSSh calculations should thus primarily be due to a more correct description of the electronic structure rather than being due to accidental error cancellations of both model and method errors. Whether this ability to describe the resting state FeMoco well, carries over to describing the relative stability of hydrides and protonation of a metal-carbide is not as clear. We note though that the TPSSh functional has been shown to be a reliable functional for many problems in inorganic and bioinorganic chemistry giving both good structures^75^ and reaction energies.^76^ It appears most likely to us that the higher HF exchange in hybrids such as B3LYP, BHLYP and M06-2X is responsible for introducing severe artifacts in the basic electronic structure of FeMoco, that would carry over to the description of all redox states. Results from these functionals, such as a tendency to favour protonated carbides (not found with functionals that describe the E_0_ structure well), should therefore be regarded with high suspicion, in our view, as 20% or higher HF exchange appears to destabilize metal–ligand and metal–metal bonding in FeMoco (resulting even in a wrong Mo oxidation state) that is arguably crucial to its reactivity.

In our view, the relative energy comparison at the TPSSh level is at this point the most reliable as only this level of theory describes the electronic structure of the cofactor accurately in the resting state. This comparison indicates E_4_ models **j–o** as the most energetically favourable E_4_ models. We note that the stability of such open sulfide-bridge models is for the most part also found at the non-hybrid level of theory (TPSS) while the higher HF exchange hybrids predict a very different energy landscape. Geometry optimizations with these hybrid functionals (B3LYP, M06-2X) would no doubt further change the energy landscape of E_4_ models (likely further stabilizing protonated carbide models), however, this would not lead to any useful comparison, in view of the aforementioned electronic structure artifacts.

Thus, models **j–o** appear to be viable choices for the E_4_ state of FeMoco. We hesitate to distinguish further between these models based on their energetics. There is ample uncertainty regarding the reliability of relative energies with our DFT level of theory but it is also worth noting that the E_4_ state is thermodynamically not stable with respect to H_2_ formation (that leads back to the E_2_ state) according to experiments. The E_4_ state must then be a state that is kinetically trapped behind a barrier, and not necessarily at the lowest energy configuration (prior to H_2_ dissociation). The **E_4_-n** model is the lowest in energy according to our energetic comparison but this is a state where H_2_ has already formed, bound to Fe_2_ with a Kubas-type metal-dihydrogen geometry. Our calculations indicate an almost barrierless H_2_ dissociation from this state (a path leading to the E_2_ redox level) and this H_2_ formation (involving hydrides) would thus occur without N_2_ involvement which is inconsistent with isotope-substitution experiments. Those experiments showed that N_2_ is necessary for H_2_ formation *via* the hydrides (criterion iii from Section A.). Additionally, the **E_4_-n** model does not feature bridging hydrides, at odds with the structural interpretation of the ENDOR studies (criterion i from Section A.). The **E_4_-n** state, while energetically favourable, may thus be a state never formed under experimental conditions, it most likely represents an alternative path towards E_4_→E_2_ relaxation, but likely one that would not be seen experimentally.

**Fig. 4:**
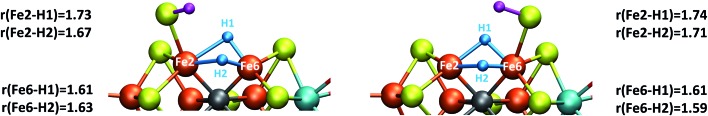
Close-up view of the hydride structures in **E_4_-l** (left) and **E_4_-o** (right) with Fe–H bond lengths (Å) indicated.

If we focus on models that seem broadly consistent with the ENDOR proposal of two almost chemically equivalent bridging hydrides then models **E_4_-l** and **E_4_-o** (see Fig. 4) appear to be the most appealing models as these models feature two almost equivalent hydrides with considerable bridging character. These two models are also among the most thermodynamically stable models according to our TPSSh protocol and there is very likely a kinetic barrier towards H_2_ formation involving these hydrides that could be affected by N_2_ binding, making a reductive elimination step possible.

On the other hand, model **E_4_-e** has been previously suggested by Hoffman and coworkers to be a structure that is consistent with the ENDOR data. In a very recent study,^25^ a case was made for this model being in the best agreement with even higher resolution ENDOR data while a structure similar to model **E_4_-o** was considered less likely. This comparison was based on calculated hyperfine tensor orientations with a simplified point-dipole approximation where the metal ions are assumed to all be high-spin and to behave as localized “spherical balls of spin”. The latter seems questionable, in our view, considering the strong delocalization of electrons seen in calculations of FeMoco and we also note that calculated spin populations of almost all E_4_ models (including **E_4_-l**, **E_4_-o** and **E_4_-e**, see Tables S2–S5†) show reduced spin populations on some Fe ions, calling into question the high-spin nature of all Fe ions in the E_4_ models. A direct calculation of the ^1^H hyperfine tensors of the hydrides of these models *via* multireference wavefunction theory may in fact be required to confidently tell apart models based on the hyperfine tensors. Such calculations are unfortunately still out of reach but may become possible in the near future. Our QM/MM calculations of **E_4_-e** do not suggest the state as a thermodynamically favourable model for the E_4_ state compared to open-sulfide bridge models (with no functional tested). Further studies will be required to determine whether such a model could be favourable under some conditions or even whether such a model represents a kinetically trapped state.

The energetically favourable **E_4_-l** and **E_4_-o** models (at TPSSh level of theory), featuring bridging hydrides (in agreement with ENDOR analysis), will be studied further in the next section. The energy surface and barriers that connects all of the states **j–o** will be reported on later; at present it is not clear whether all of these states are accessible to each other and this requires careful mapping of the minimum energy paths between them.

### D. Molecular and electronic structure of **E_4_-l** and **E_4_-o**

The two models **E_4_-l** and **E_4_-o** both feature the same bridging hydride structure with the hydrides between Fe_2_ and Fe_6_ but the models differ in the position of the terminal sulfhydryl group (Fe_2_*vs.* Fe_6_) derived from sulfide S2B (Fig. 4). Sulfide S5A is also protonated in these models but remains bridging. The two hydrides in these models are mostly but not completely bridging between Fe_2_ and Fe_6_. For model **E_4_-l**, both hydrides are slightly more associated with Fe_6_ (Fe_6_–H distances of 1.61 and 1.63 Å) than Fe_2_ (Fe_2_–H distances of 1.73 and 1.67 Å) and for model **E_4_-o**, we see similarly stronger binding to Fe_6_ as well, with Fe_6_–H distances of 1.61 and 1.59 Å and Fe_2_–H distances of 1.71 and 1.74 Å. The coordination of these two new ligands to Fe_2_ and Fe_6_ change the geometry of these Fe ions from approximate tetrahedral to five- and six-coordinate geometries (distorted octahedral geometries), which naturally has a pronounced effect on the electronic structure of both Fe ions. In fact, the electronic structure of the two models reveal a change in the local spin state of both Fe ions in the lowest energy solution found for both, BS-147. With hydrides known to act as strong-field ligands and the change in four-coordinate local tetrahedral geometry to five or six-coordinate geometry this is perhaps not surprising. The change in local spin state is revealed in the change of spin population at Fe_2_ and Fe_6_ (from >3 to 2.4–2.5, see Table S6† for spin populations) and the localized orbitals reveal more clearly spin-pairing occurring at Fe_2_ and Fe_6_. The electronic structure appears consistent with intermediate spin *S* = 3/2 Fe(iii) ions but this will require further study. In this context it is worth noting that such a double-hydride bridging geometry between two Fe ions has precedent in the form of synthetic dimeric Fe compounds from the groups of Peters^77^ and Holland^78^ though these synthetic compounds have a planar Fe–H–Fe–H geometry.

A bridging hydride structure, with two hydrides between Fe_2_ and Fe_6_, has also been discussed by Einsle and coworkers as a possible E_4_ structure.^33^,^79^ However, that structure assumed an absent sulfide, in line with the hypothesis that the sulfide S2B leaves to open up a binding site, as crystal structures have shown that the sulfide can be displaced by CO or NH/OH.^12^,^33^ Importantly, in our E_4_ models, the S2B sulfide is protonated and remains present as a terminal sulfhydryl group on either Fe_2_ or Fe_6_. Dance has recently explored the thermodynamic feasibility of completely dissociating S2B in the form of SH^–^ or H_2_S and found it not to be favourable^80^ and our own preliminary results suggest this as well. It is presently not clear under what conditions (or when in the cycle) complete sulfide removal from the cofactor (as shown by the crystal structures) occurs.

Based on the similarity of models **E_4_-l** and **E_4_-o** in terms of structure and energy, we cannot easily distinguish between them. Both appear reasonable candidates for the E_4_ state; only one of them may be formed under turnover conditions, however, and this may depend on the precise way that protons and electrons get introduced into the active site. Unfortunately, these mechanisms are not well established.

### E. Dinitrogen binding to **E_4_-l** and **E_4_-o**

With the change in coordination by introduction of bridging hydrides, one of the Fe ions in each state is coordinatively unsaturated with respect to a six-coordinate octahedral geometry. This makes N_2_ binding to Fe_6_ (in **E_4_-l**) or Fe_2_ (in **E_4_-o**) a feasible scenario. In fact the synthetic Fe(ii)–(μH)_2_–Fe(ii) dimer by Peters and coworkers featured a bound N_2_ ligand on one of the Fe ions and 1-electron reduction to give a Fe(ii)–Fe(i) dimer resulted in a large increase in the binding affinity of a second N_2_.^77^ There is thus precedent for a similar bridging-hydride dimer structure to be capable of favorable N_2_ binding.

**Fig. 5:**
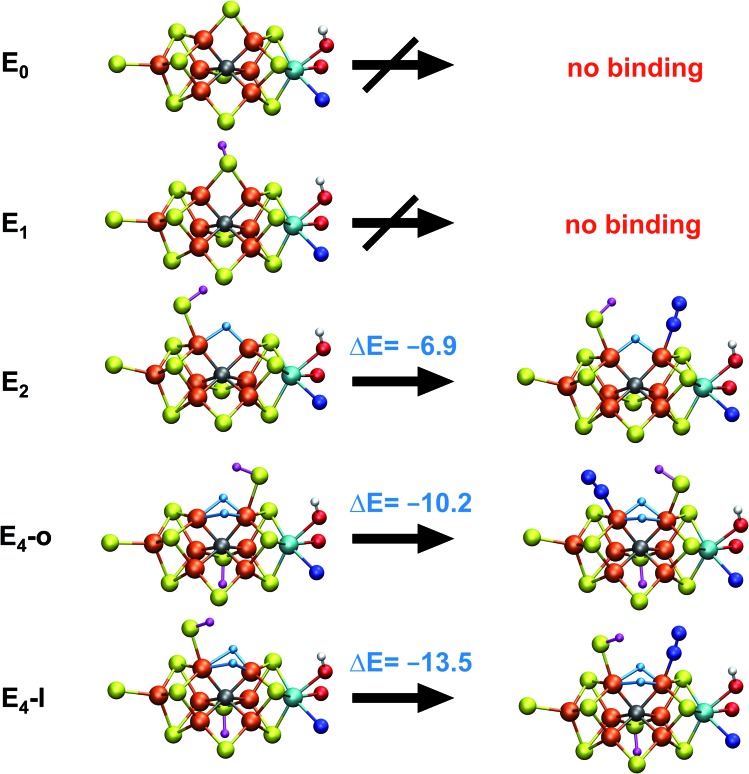
N_2_ binding of **E_4_-l** and **E_4_-o** models compared to models of earlier redox states according to QM/MM calculations. The **E_1_** model features a 1-electron reduced FeMoco (*M*_S_ = 2) with S2B protonated while the **E_2_** model (BS-235, *M*_S_ = 3/2) is analogous to the **E_4_-l** model with a bridging hydride between Fe_2_ and Fe_6_ and a terminal sulfhydryl group on Fe_2_. N_2_ binding energies (kcal mol^–1^) are relative to free N_2_ and are electronic energies. Accounting for translational entropy, (10.7 kcal mol^–1^, based on gas phase statistical mechanics), would decrease the binding energies by that amount.

We explored N_2_ binding to the empty coordination site at Fe_6_/Fe_2_ for both **E_4_-l** and **E_4_-o** models to give N_2_-bound models **E_4_-l-N_2_** and **E_4_-o-N_2_** (shown in Fig. 5). N_2_ binding is found to be thermodynamically favourable (w.r.t. free N_2_) for both **E_4_-l** (bound to Fe_6_) and **E_4_-o** (bound to Fe_2_) states, with an electronic binding energy of Δ*E* = –13.5 kcal mol^–1^ for the **E_4_-l-N_2_** state and a slightly weaker binding of Δ*E* = –10.2 kcal mol^–1^ is found for the **E_4_-o-N_2_** state. We note that accounting for translational entropy (10.7 kcal mol^–1^ based on gas phase statistical mechanics) would reduce these electronic binding energies by that amount, leading to slightly endothermic 0.5 kcal mol^–1^ binding for **E_4_-o** and exothermic 2.8 kcal mol^–1^ for **E_4_-l**. Different broken-symmetry solutions (BS-235, BS-346, BS-247 and BS-147) were tested for the N_2_-bound states **E_4_-l-N_2_** and **E_4_-o-N_2_** that turned out to be crucial, as the energies of different states differed by up to 14 kcal mol^–1^ (see ESI, Table S6†), with some BS states not showing favorable N_2_ binding. For **E_4_-l-N_2_** and **E_4_-o-N_2_** models, the BS-235 and BS-346 solutions were lowest in energy, respectively. We note that these results are in sharp contrast with a recent study by Raugei *et al.*^24^ that found N_2_ binding (in a bridging geometry between Fe_2_ and Fe_6_) to be endothermic by 5 kcal mol^–1^ (Δ*E*).

N_2_ binds end-on to Fe_6_/Fe_2_ in these **E_4_-l-N_2_** and **E_4_-o-N_2_** structures resulting in an unusual distorted octahedral geometry at the participating Fe. This favorable binding of N_2_ to a FeMoco redox state is notable; N_2_ will unsurprisingly not bind to FeMoco at the E_0_ redox level in our calculations (no minimum found) but the same applies to a 1-electron reduced state. Our model for the E_1_ redox state (one of two favoured models from a recent joint EXAFS-QM/MM study^15^) involves an added electron to the [MoFe_3_S_3_C] sub-cubane and a protonated S2B sulfide bridge (multiple BS states with *M*_S_ = 2 were calculated). Even when the E_1_-N_2_ optimization is started from a structure with a terminal sulfhydryl group bound to Fe_2_ and N_2_ in close proximity to Fe_6_, then N_2_ spontaneously dissociates. Thus neither 1-electron reduction of the cofactor or protonation of the sulfide bridge alone is sufficient for favorable N_2_ binding to occur at the cofactor. N_2_ will also not bind favorably to other Fe ions in the **E_4_-l** model; while a stable minimum is found when N_2_ is placed at Fe_7_, the binding is endothermic (∼5.5 kcal mol^–1^).

These results suggest that the hydride ligands at Fe_2_ and Fe_6_ in **E_4_-l** and **E_4_-o** are responsible for the favorable formation of the N_2_-binding states, **E_4_-l-N_2_** and **E_4_-o-N_2_**, likely due to the unique ligand-field now found at these Fe ions. In support of this, we have also calculated a model for the E_2_ state of FeMoco, analogous to **E_4_-l** (with a sulfhydryl group at Fe_2_) but with only one bridging hydride between Fe_2_ and Fe_6_. This state (BS-235 and *M*_S_ = 3/2) interestingly features favorable N_2_ binding (–6.9 kcal mol^–1^), about half of the binding energy to the **E_4_-l** state. The calculated N_2_ binding of this E_2_ model may, however, not be enough to overcome entropy and implies that the presence of two hydrides at the same Fe may be required for favorable dinitrogen binding.

Electronic structure aspects of dinitrogen binding of FeMoco will be explored in more detail in a future study. Our current interpretation of the electronic structure behind the favorable Fe_6_–N_2_ binding in the **E_4_-l**→**E_4_-l-N_2_** step is that the low-spin configuration found at Fe_6_ in **E_4_-l-N_2_** (spin population of –0.46) is a vital aspect of the N_2_ binding. The localized orbital analysis of Fe_6_ in **E_4_-l-N_2_** reveals that this iron can be interpreted as a low-spin octahedral *S* = 1/2 Fe(iii) ion with doubly occupied d_*xz*_ and d_*yz*_ orbitals. This low-spin configuration must arise *via* the strong-field hydride ligands as well as due to the N_2_ ligand. The double occupation of the d_*xz*_ and d_*yz*_ orbitals should allow increased backbonding to the N_2_ π* orbitals and the localized orbitals reveal some N_2_ character in these orbitals (see Fig. S8†). Taken together, the results in this section imply that a change from a high-spin to a low-spin Fe configuration may be vital to N_2_ being able to bind to FeMoco in the first place. The same argument holds for the **E_4_-o-N_2_** model.

While the N_2_ ligand binds more strongly to Fe_6_ in our **E_4_-l-N_2_** structure than to Fe_2_ in the **E_4_-o-N_2_** structure, the difference is not large enough to confidently tell the two scenarios apart. The N_2_ ligand has a slightly elongated N–N bond length of 1.109 and 1.112 Å (for **E_4_-l-N_2_** and **E_4_-o-N_2_** respectively), a shift of 0.014/0.017 Å compared to free N_2_ at the same level of theory. The shift in N–N vibrational frequency (compared to free N_2_ at our level of theory) of 172 cm^–1^ (**E_4_-l-N_2_**) and 202 cm^–1^ (**E_4_-o-N_2_**) also indicates weak N_2_ activation for both states.

### F. Reductive elimination

As is now well established from experiments, H_2_ is eliminated from the E_4_ state as N_2_ binds, *via* a reductive elimination mechanism involving the hydrides.^4^,^19^–^21^ Due to the proximity of the two hydrides in the E_4_ and E_4_-N_2_ models discussed in the previous chapters, one can imagine how such a reductive elimination step could proceed from our models. Isotope substitution experiments indicate that H_2_ evolution *via* reductive elimination can only proceed in the presence of N_2_. Experiments performed in the absence of N_2_, indicate that a regular hydride-proton reaction is responsible for the H_2_ formed from the E_4_ state (leading to the unproductive E_4_→E_2_ side-reaction). This implies that as N_2_ binds to the E_4_ state, either the H_2_ formation *via* reductive elimination is favoured thermodynamically over a hydride-proton reaction or it is kinetically favoured by lowering of the barrier for reductive elimination (relative to a hydride-proton reaction).

A future study will explore in detail the reaction barriers for H_2_ formation *via* both reductive elimination and hydride-proton mechanisms from our E_4_ models. However, there is a simple argument in favour of reductive elimination over a more normal hydride-proton reaction that applies to both **E_4_-l-N_2_** and **E_4_-o-N_2_** models. The simplest hydride-proton reaction would involve combining one of the bridging hydrides with the proton from a sulfhydryl group (the closest accessible proton). The required deprotonation of the terminal sulfhydryl group (to act as proton donor) would be highly unfavoured as the terminal deprotonated sulfide is prevented from recombining with Fe_6_/Fe_2_ to reform the sulfide-bridge due to the presence of the other hydride in this position (in fact, calculations indicate such a step to be uphill by 15.1 kcal mol^–1^). The dinitrogen ligand would furthermore block a hydride-proton pathway involving the hydroxy proton from the Mo-bound homocitrate and possibly other proton donors. The molecular structures of the **E_4_-l-N_2_** and **E_4_-o-N_2_** models thus appear to offer an intuitive explanation for why the reductive elimination step can only occur in the presence of N_2_. Without the N_2_ ligand (*i.e.* in the absence of N_2_ gas), a hydride-protonation reaction (*i.e.* a E_4_→E_2_ step) should be more likely to occur, probably due to a lower barrier of a hydride-proton reaction compared to a hydride–hydride reaction. This could occur *e.g.* by hydride protonation *via* the hydroxy group on Mo-bound homocitrate or *via* the sulfhydryl group (and a possible transformation of the remaining bridging hydride to a terminal hydride and reformation of the sulfide bridge). With the N_2_ ligand present, however, reductive elimination could be the only favoured H_2_ evolution pathway.

**Fig. 6:**
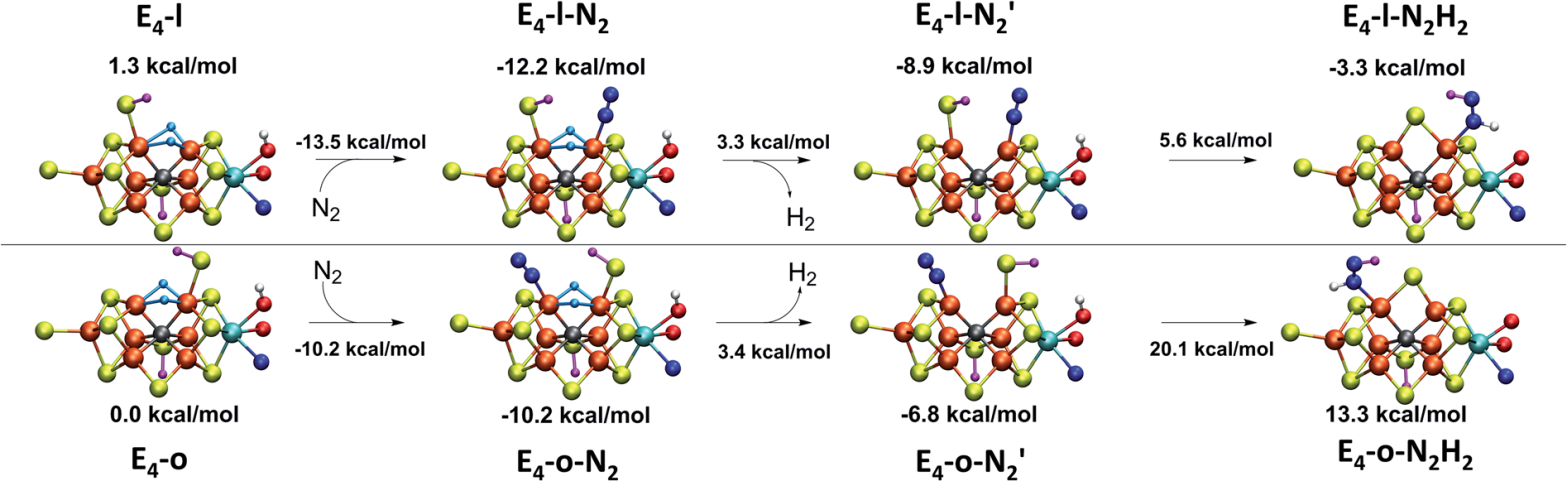
N_2_ binding, reductive elimination and N_2_ protonation reactions for the **E_4_-l** and **E_4_-o** structures. State energies are relative to the **E_4_-o** model. A reductive elimination of H_2_*via* the bridging hydrides releases 2 electrons to give a doubly-reduced cofactor in states 
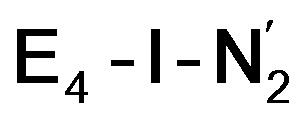
 and 
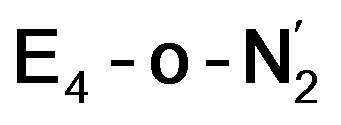
. A possible subsequent protonation step of N_2_*via* the sulfhydryl group and hydroxy group of homocitrate, reforms the sulfide bridge between Fe_2_ and Fe_6_ to form diazene-bound intermediates **E_4_-l-N_2_H_2_** or **E_4_-o-N_2_H_2_** at either Fe_2_ or Fe_6_.

This reductive elimination step involving two hydrides (formally storing four electrons) releases two electrons in the form of H_2_ and makes two electrons now available to the cofactor in states we call 
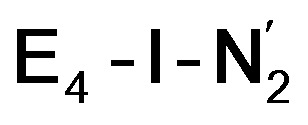
 and 
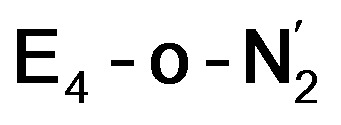
 (see Fig. 6). Our calculations predict an electronic energy change (Δ*E*) of +3 kcal mol^–1^ uphill. While this step is predicted to be mildly endothermic according our calculations, this result is not in strong disagreement with experiment. In fact, kinetic studies show this step to be reversible,^81^ meaning the step is close to thermoneutral. Additionally, the translational entropy contribution to the free energy would favour the elimination of H_2_. We have opted for not adding gas phase translational entropy corrections (–8.4 kcal mol^–1^ for H_2_ elimination) to our energies as the entropic contribution could be more complicated for a complex condensed phase macromolecular system such as nitrogenase (as has been discussed by Reiher and coworkers^82^), however, the entropy contribution would likely be the same magnitude as the gas phase value. The fact that the 
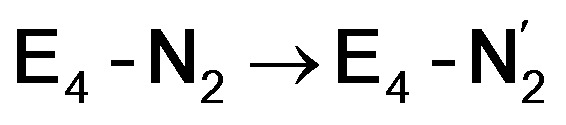
 step is predicted to be close to thermoneutral (Δ*E*) according to our calculations is due to the nature of the reductive elimination. A regular H_2_ formation step should be quite exothermic (our calculations for hydride-proton reactions for an E_4_ → E_2_ predict exothermicity of ∼ –20 kcal mol^–1^). The lack of strong exothermicity for 
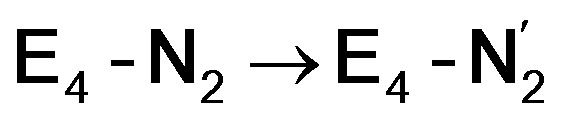
 step stems from the fact that the metal cofactor is reduced (by 2 electrons) during the reaction, as previously the four electrons were stored in the form of the hydrides and two leave in the form of H_2_. This rather unfavorable two-electron reduction of the metal cofactor may thus be offset *via* the exothermicity of the H_2_ formation.

The molecular and electronic structure of the 
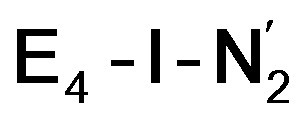
 and the 
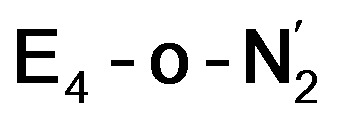
 states is thus of interest as the metal ions of the cofactor are now more reduced than before and the structures lack the hydrides that previously helped bind N_2_. The local Fe geometries of the Fe_2_/Fe_6_ ions in 
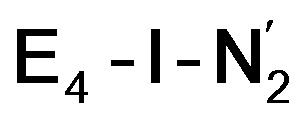
 and 
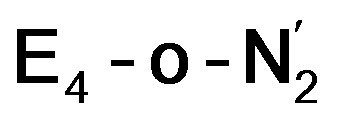
 are unusual. A terminal sulfhydryl group is still present on Fe_2_ or Fe_6_. Attempts to reform the sulfide bridge (with sulfide still protonated) were not successful as the system returns to a geometry with the terminal sulfhydryl group. Clearly, the distorted geometries at Fe_2_/Fe_6_ are stable, though this is presently not well understood. Using the *τ*_4_ and 
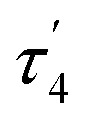
 structural metrics^83^,^84^ for 4-coordinate compounds, we find that the Fe_2_ and Fe_6_ in both 
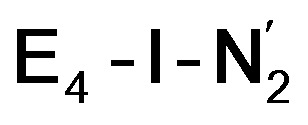
 and 
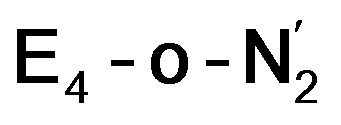
 models are about halfway between a tetrahedral geometry and a seesaw geometry (see ESI, Tables S7 and S8,† for calculated *τ*_4_ and 
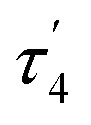
 parameters for all Fe ions in multiple E_4_ models). We note that such geometries have previously been found in DFT calculations of FeMoco.^85^,^86^

Analysis of the electronic structure suggests that the distorted N_2_-bound Fe_2_ ion in 
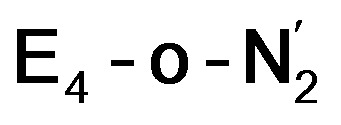
 can be described as an *S* = 1 Fe(ii) ion. However, remarkably, the distorted Fe_6_ ion in 
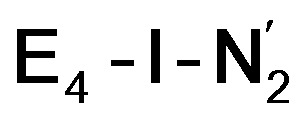
 best fits the description of an *S* = 3/2 Fe(i) ion according to the localized orbitals (see ESI, Fig. S9†), revealing a stark difference between the two states and the nature of the different binding site. Both 
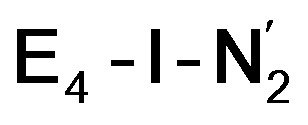
 and 
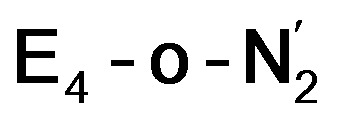
 states feature doubly occupied d_*xz*_ and d_*yz*_ orbitals that likely account for the favorable dinitrogen binding *via* metal backbonding (similarly to the **E_4_-l-N_2_** and **E_4_-o-N_2_** models despite the absence of hydrides).

While distinguishing between the **E_4_-l** and **E_4_-o** pathways is not quite clear based on the calculated energies, it is tempting to attribute the presence of Fe(i) in 
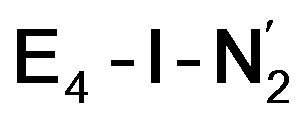
 as a potentially important aspect of the mechanism when going forward. The interpretation of the reductive elimination is then that the electrons in the reductive elimination step are used to reduce the N_2_-binding Fe_6_ ion to an Fe(i) ion. Compared to the resting state E_0_, the mixed-valent delocalized Fe_6_(2.5)–Fe_7_(2.5) pair has then been reduced to a pair of localized Fe_6_(i) and Fe_7_(ii) ions. Thus, the reductive elimination step could be imagined as a specific mechanism towards stabilizing an Fe(i)–N_2_ species without going to the strongly negative potentials such as those required for mononuclear complexes. Indeed, low-valent mononuclear iron complexes from Jonas Peters and coworkers have been found to act as catalysts for N_2_ reduction; this is accomplished *via* the use of strong reductants (KC_8_) to access low (Fe(i), Fe(0) and Fe(–i)) oxidation states that bind and activate N_2_.^87^–^89^ Reductive elimination could thus enable reduction of an Fe ion to Fe(i) while using the same low potential of the Fe protein.

The N_2_ ligand in 
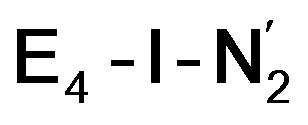
 and 
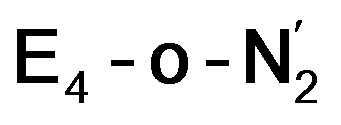
 is only weakly activated as seen in a small increase in N–N bond length of 0.021 and 0.023 Å for 
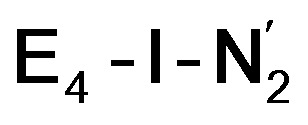
 and 
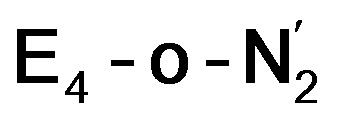
 respectively, when compared to free N_2_ and by the decrease in N–N vibrational frequency of 242 and 255 cm^–1^ for 
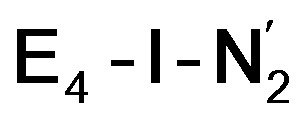
 and 
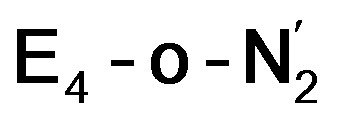
 respectively, when compared to free N_2_. The N_2_ activation is greater though than in the **E_4_-l-N_2_** and **E_4_-o-N_2_** models (N–N bond length shifts of 0.014 and 0.017 Å and frequency shifts of 172 cm^–1^ and 202 cm^–1^). A high-spin Fe(i)–N_2_ complex was first synthesized by Harman and coworkers.^90^ Unlike the 
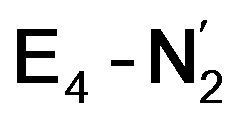
 states proposed here, the synthetic Fe(i)–N_2_ complex has *C*_3v_ symmetry and the dinitrogen activation is stronger with a N–N stretching frequency shift of 372 cm^–1^ compared to free N_2_.

While activation of the N_2_ ligand in the in 
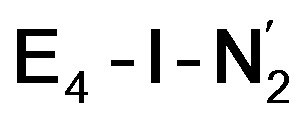
 and 
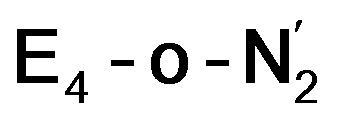
 states is thus still considered weak, and protonation might be considered unlikely, we tested simple protonation reactions by transferring nearby protons to the nitrogen atoms of the ligand. Two possible protonation agents seem likely, one being the terminal sulfhydryl group on Fe_2_/Fe_6_ and the hydroxy group of homocitrate another. The hydroxy proton on homocitrate was found to be present in the resting state FeMoco according to a previous QM/MM study by us^13^ and by Cao *et al.*^91^ In this context it is of note that homocitrate is experimentally known to be important for dinitrogen reduction.^92^ Transferring a proton from the hydroxy group to either the distal or proximal nitrogen of either 
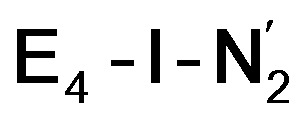
 or 
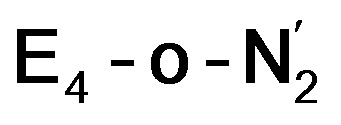
 resulted in high energy intermediates of 34.3–36.3 kcal mol^–1^. If a double proton-transfer step is calculated, however, using both the sulfhydryl group and the hydroxy group, we get lower-energy diazene intermediates **E_4_-l-N_2_H_2_** and **E_4_-o-N_2_H_2_** instead. While the **E_4_-l-N_2_H_2_** diazene intermediate is predicted to be higher in energy than the 
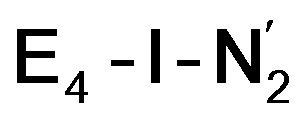
 state by 5.6 kcal mol^–1^, it is interestingly much more favorable than the **E_4_-o-N_2_H_2_** intermediate (uphill by 20.1 kcal mol^–1^), demonstrating very different reactivity of the Fe sites in the two models. Furthermore, the 
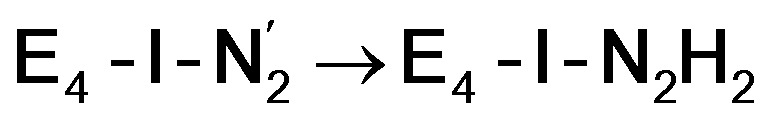
 pathway, being in close proximity to the hydroxy group appears more plausible for direct proton transfer. These results indicate that protonation of the N_2_ ligand may not occur until later in the cycle (*e.g.* in the E_5_ redox state) or possible *via* another protonation mechanism not considered here.

Experimentally, *via* a quench-cryoannealing relaxation protocol and varying H_2_/N_2_ concentrations, a reaction intermediate was shown to accumulate following reductive elimination, at the same redox level as E_4_.^81^ While this intermediate is denoted as E_4_(2N2H) by Hoffman and coworkers and discussed as “*a state in which FeMo-co binds the components of diazene, which may be present as N*_*2*_*and two [e*^*–*^*/H*^*+*^*] or as diazene itself*”^81^ EPR and ENDOR studies of this same intermediate^93^,^94^ showed ^15^N hyperfine coupling (using ^15^N_2_) but no hyperfine coupling from hydrogens was seen. A single ^15^N hyperfine signal was found, suggesting end-on binding which fits well with our 
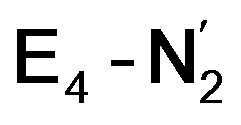
 models. A recent EPR/ENDOR study of synthetic mononuclear Fe–N_2_ compounds by Peters and coworkers of states featuring either a single and double protonated N_2_ ligand: Fe–N_2_H and Fe–N_2_H_2_ (distal protonation) revealed a clear hyperfine signal from the hydrogens.^95^ These experiments may thus be an indication that the experimental dinitrogen-bound intermediate, “E_4_(2N2H)” is still unprotonated. Based on our computational modelling, it is possible that the experimental intermediate “E_4_(2N2H)” could correspond to the 
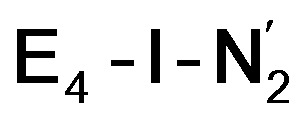
 model (or possibly 
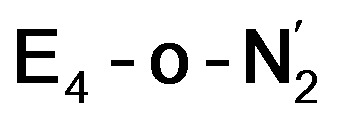
) rather than a diazene-bound intermediate like **E_4_-l-N_2_H_2_**/**E_4_-o-N_2_H_2_**. An alternative explanation is that the hyperfine couplings from the hydrogens in a diazene intermediate may be too weak to be measured. Additional spectroscopy is required to clarify the nature of this intermediate.

## Conclusions

We have presented QM/MM calculations of possible models for the E_4_ state of nitrogenase and how dinitrogen can bind to some of these models. In view of the thermodynamic stability of our model for the E_4_ state and the favourable dinitrogen binding we have presented, our work looks promising as a step towards a mechanistic understanding of biological dinitrogen reduction. Questions remain about the reliability of calculated reaction energies for this complicated cofactor and a stronger connection to experiment would be much desired, requiring more spectroscopy on nitrogenase intermediates.

In this context, we should note that a recent ENDOR study^25^ combined with calculations has presented evidence in favour of another model (model **e** in article) as the structure of the E_4_ state. Our QM/MM calculations of that model suggest it to be higher in energy than all open-sulfide bridge models (with all calculated functionals) and this model has not been found to bind dinitrogen favorably.^24^ Additional spectroscopic data on the E_4_ state to sort out this disagreement would therefore be desirable.

Our proposed mechanism for binding of dinitrogen and reductive elimination suggests a role for many of the components of the complicated cofactor of nitrogenase. The size of the cofactor and the nature of the fused iron–sulfur double-cubane may play a primary role of favourably accepting electrons and storing them as hydrides at the same potential as provided by the Fe protein; the stability of the hydride geometries being facilitated by a labile sulfide bridge between cubanes. Furthermore, our calculations suggest the strong-field nature of the hydrides to aid the binding of dinitrogen by a local high-spin→low-spin electronic structure change at Fe_6_ or Fe_2_. The reductive elimination step then maintains the low-spin structure while doubly reducing the cofactor and partially activating the N_2_ ligand. The carbide may play a role in tuning the redox potential for the reduction steps but it may also play a role in either binding or activation of N_2_. The carbide being approximately *trans* to the N_2_ ligand in our 
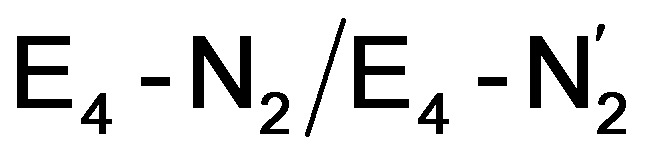
 structures, suggests a link to the model chemistry of Peters and coworkers^87^,^88^ where carbide and boride-containing mononuclear trigonal bipyramidal Fe complexes were found to be active catalysts for dinitrogen reduction. Similar to the model compounds, the carbide in FeMoco may aid in pushing electron density into the π-accepting orbitals of the N_2_ ligand. The molybdenum ion likely also has an effect on the redox potential of the cofactor (the redox potential can in fact be tuned by heterometal substitution as known by synthetic [XFe_3_S_4_] chemistry where X = Mo, V, W^96^,^97^). We speculate that the role of the molybdenum may also be vital in stabilizing the N_2_-bound Fe(i) ion in 
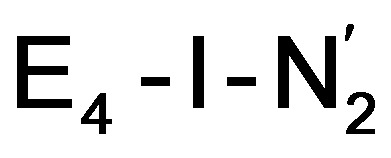
, formed after the reductive elimination step, making the electrons available for partial activation of the N_2_ ligand. The homocitrate ligand likely plays a role in protonation steps and the proton on the Mo-bound alcohol group of homocitrate is in an ideal position to protonate the N_2_ ligand when bound to Fe_6_ in the 
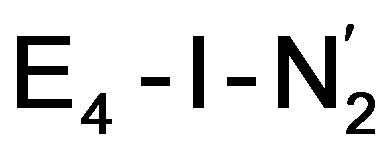
 state; it may also help deliver protons in the early redox states (becoming protonated sulfides or hydrides).

Finally, we emphasize that this work presents only a preliminary mechanism for dinitrogen binding to FeMoco that relies primarily on calculated energies with density functional theory approximations (where a large functional dependency is seen) and where only a small part of chemical space has been explored. More experimental data on the E_4_ and E_4_-N_2_ states are urgently needed to further constrain the mechanistic possibilities of FeMoco as well as to help benchmark the theoretical methodology employed. The precise way in which the N_2_ ligand is activated for protonation is also not clear from our results. At the very least this computational study has presented falsifiable ideas about the mechanism of biological nitrogen reduction that can be confirmed or ruled out by suitable experiments.

## Conflicts of interest

There are no conflicts to declare.

## Supplementary Material

Supplementary information

Supplementary information
