# Supplementary material for: A model for dinitrogen binding in the E4 state of nitrogenase
Source: Chem Sci. 2019 Oct 15;10(48):11110–24. doi: 10.1039/c9sc03610e (PMC7069239; doi:10.1039/c9sc03610e)
Supplement: Supplementary file 1 [file SC-010-C9SC03610E-s001.pdf]

Electronic Supplementary Material (ESI) for Chemical Science.
This journal is © The Royal Society of Chemistry 2019

A model for dinitrogen binding in the E_4 state of nitrogenase

Albert Th. Thorhallsson,[1,2] Bardi Benediktsson[1] and Ragnar Bjornsson[1,2]*
[1]Science Institute, University of Iceland, Dunhagi 3, 107 Reykjavik, Iceland.
[2] Department of Inorganic Spectroscopy, Max-Planck-Institut für Chemische Energiekonversion, Stiftstrasse 34-36, 45470 Mülheim an der Ruhr (Germany).
* E-mail: ragnar.bjornsson@cec.mpg.de

Details of the QM/MM modelling

The MoFe protein of the molybdenum nitrogenase is a large $\alpha_2\beta_2$ heterotetramer structure and is comprised of over 2000 amino acid residues. The model used in this work is derived from a previous study of ours of the resting state of the iron-molybdenum cofactor (FeMoco) of MoFe protein[1]. The details of the MM and QM/MM model setup is summarized here:

Model preparation

For the initial classical (MM) model the program GROMACS[2-4] was used with the protein described by a CHARMM36 forcefield[5]. Atomic charges for FeMoco and the P-cluster were derived from natural population analysis of the clusters at the BP86/def2-TZVP level of theory. Lennard-Jones parameters were assigned to the metal-sulfur clusters similarly using the CHARMM SM atom type for inorganic sulfides. Atomic charges and Lennard-Jones parameters for homocitrate are based on parameters available for citrate[6]. Parameters for imidazole molecules present in the crystal structure were taken from CGenFF[7]. Cysteine residues connected to the metal clusters were modelled as deprotonated cysteine residues (CSD residues in CHARMM36). The TIP3P model was used for water residues. The 1.0 Å resolution crystal structure of molybdenum nitrogenase MoFe protein was used to prepare the model (PDB ID: 3U7Q)[8]. Two calcium ions at the interface between the two subunits of the MoFe protein were modelled as iron ions[9]. The protonation states of titratable residues were determined mostly by manual inspection i.e. looking for obvious hydrogen bond donor-acceptor pairs, and with aid from the program PROPKA[10-11]. All aspartate and glutamate were assumed to be deprotonated, except α-153^{Glu} whereas all lysine and arginine residue were determined to be protonated, except β-365^{Lys}. Histidine residues that are protonated at the epsilon nitrogen α-80^{His}, α-83^{His}, α-195^{His}, α-362^{His}, α-383^{His}, α-442^{His}, β-106^{His}, β-193^{His}, β-311^{His}, β-363^{His}, β-392^{His}, β-429^{His}, β-457^{His}, β-477^{His}, β-478^{His}, β-480^{His}. The histidine residues protonated at the delta nitrogen were α-31^{His}, α-196^{His}, α-285^{His}, α-451^{His}, β-185^{His}, β-297^{His}, β-396^{His}, β-519^{His}. Only β-90^{His} was doubly protonated at both epsilon and delta nitrogen atoms. All oxygen atoms designated as water in the crystal structure were protonated as well. After protonation, the model contains 39566 atoms. At this point, the MoFe protein is solvated in a 90*90*90 Å water box and 39 sodium ions are introduced to neutralize the overall negative charge of the protein. The final MM model contains 320829 atoms.

Water molecules and hydrogen atoms were minimized and the system then equilibrated by molecular dynamics simulations, using the canonical ensemble with a Nosé-Hoover thermostat with chain number of 4[12-15] and a simulation temperature of 300 K. Constrained and unconstrained MD simulations were tested. In the constrained MD simulation, all heavy atoms of the protein were constrained but hydrogen atoms, water molecules and counterions were free to move. At 940 ps of the MD simulation

(as can be seen in Figure S1), a snapshot of the model was taken which was used to construct the QM/MM model. In the unconstrained simulation (not used in the QM/MM modelling) the system was simulated for 5 ns with all atoms allowed to move except FeMoco and P-cluster. The average RMSD converged to 0.31 Å after ~1 ns and maintained this for the duration of the 5 ns simulation, demonstrating that the forcefield is describing the system reasonably well.

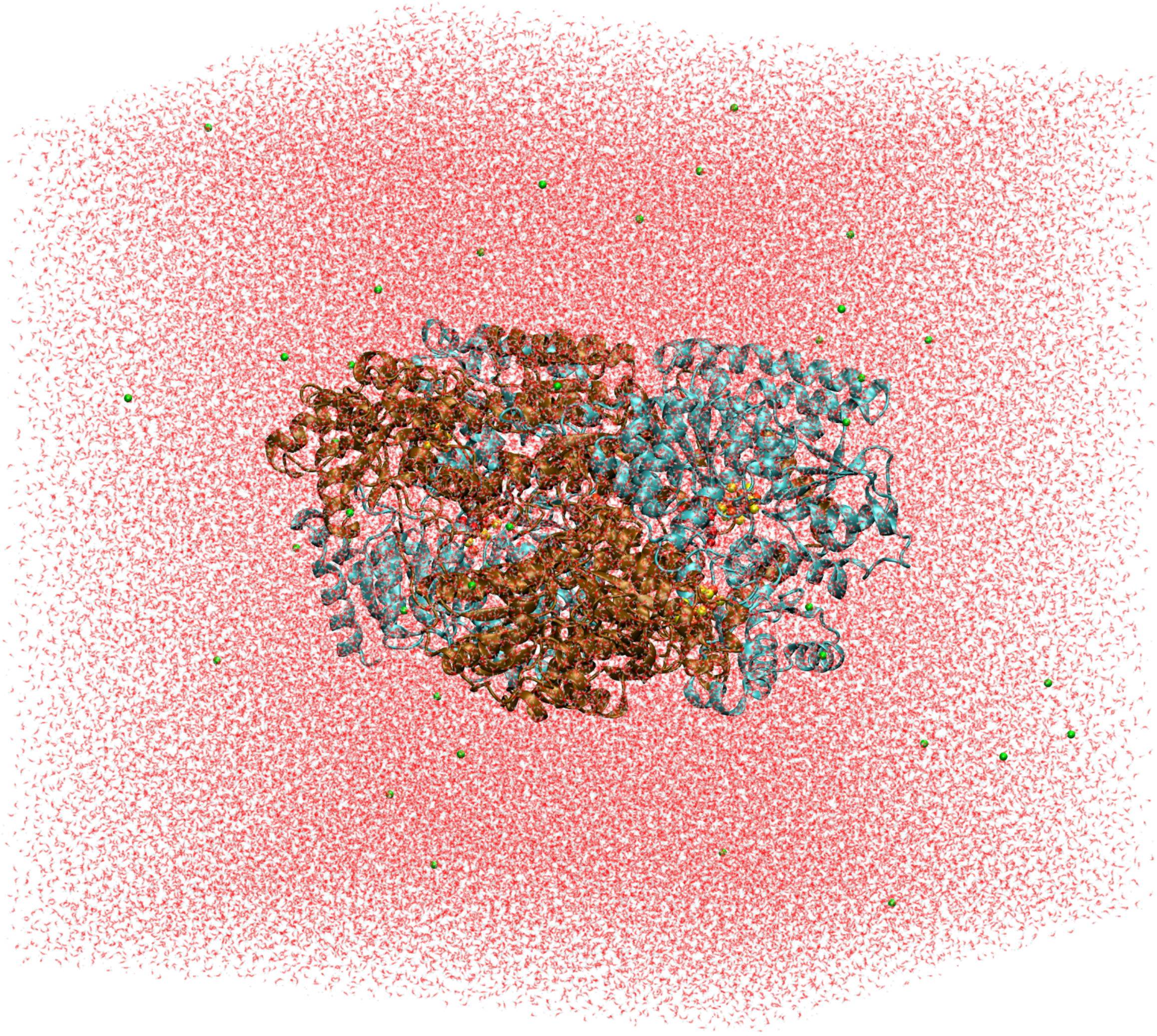

Figure S1. The 320 829 atom classical (MM) model of the MoFe protein. The protein is dissolved in a box of 93 754 water molecules and contains 39 sodium counterions.

A spherical QM/MM model (Figure S2) was prepared by cutting a sphere with a radius of 42 Å from the classical model, centered on the carbide of FeMoco in the αβ subunit, labelled as chains A and B in the crystal structure. All of chain A and chain B (i.e. a whole αβ subunit) were included in the QM/MM model as well as a portion of chain C to get a more natural cut. The residues from chain C are residues number 242, 320−326, 342− 369, 378−392, 410−419, 437−442, 458−469, 476, and 468− 523. There is also a thick water shell and the total size of the model is 36989 atoms.

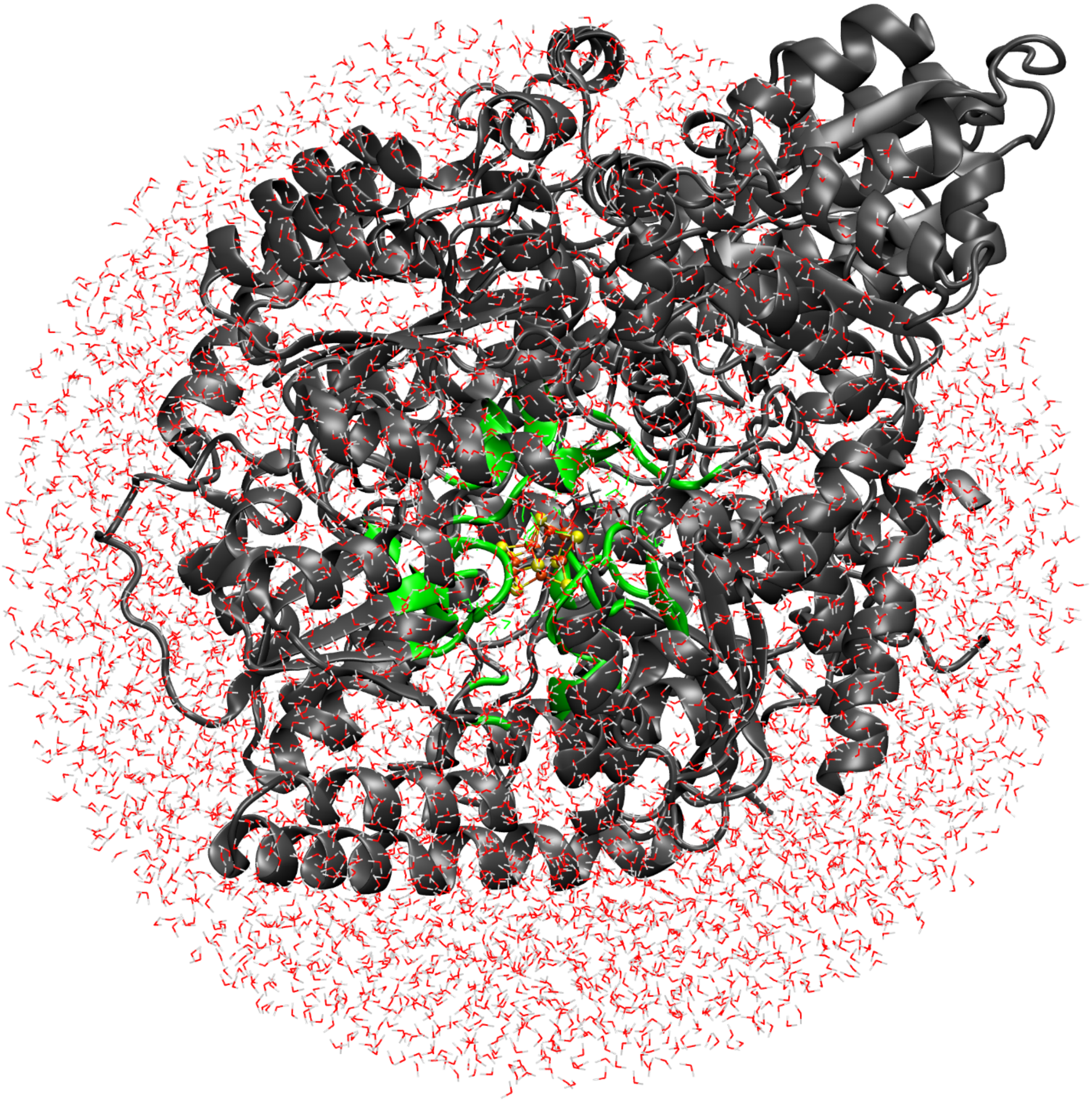

Figure S2. The QM/MM model. Colored in black is the frozen region of the protein that is described by a forcefield, whereas the red-white molecules are frozen waters also described by a forcefield. Colored in green is the active region (i.e. atoms that can move) that is either described by a forcefield or DFT (i.e. the QM region). At the center of the figure is FeMoco shown in a ball-and-stick representation.

QM/MM setup

Within the QM/MM model, we divide the model into different regions; a frozen region, an active region and a QM region. The frozen region is described at the MM level with all residues frozen at their positions from the MD snapshot.

The active region is ~1000 atoms in size (999 atoms for the E_0 model) and includes all residues within a ~11 Å radius from the central carbide of FeMoco (Figure S3). All atoms in the active region are free to move during the QM/MM geometry optimizations. Of these ~1000 atoms, most of them are described at the MM level with the exception

of the FeMoco cluster and close residues which are described at the QM level, defining a QM region.

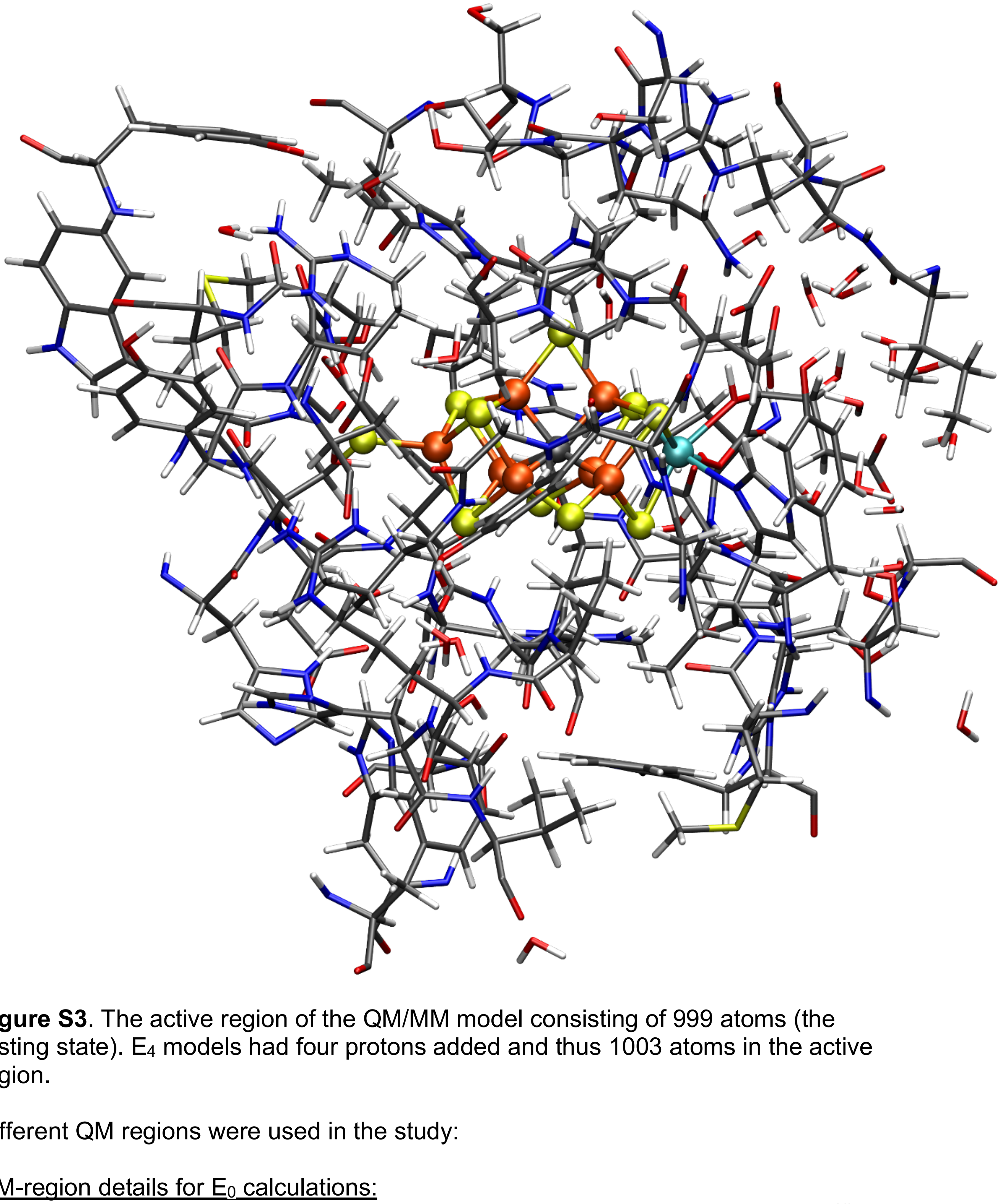

Figure S3. The active region of the QM/MM model consisting of 999 atoms (the resting state). E_4 models had four protons added and thus 1003 atoms in the active region.

Different QM regions were used in the study:

<u>QM-region details for E_0 calculations:</u>
FeMoco and the side chains of residues directly coordinating Femoco (α-442^{His}, α-275^{Cys}). Total of 54 QM atoms.

<u>QM-region details of the large 367 atom E_0 model (used in Figure S12):</u>
FeMoco and the side chain of following residues: α-70^{Val}, α-96^{Arg}, α-191^{Gln}, α-192^{Ser}, α-195^{His}, α-228^{Asp}, α-234^{Asp}, α-274^{His}, α-277^{Arg}, α-281^{Tyr}, α-359^{Arg}, α-380^{Glu}, α-381^{Phe},

α-426^{Lys}, α-427^{Glu}, α-442^{His}, α-451^{His}. We included the whole of residue α-275^{Cys} and the peptide chain part of α-354^{Tyr} -α-355^{Ile} -α-356^{Gly} -α-357^{Gly} -α-358^{Val} -α-359^{Arg} as well as the peptide chain part of β-424^{Gly} – β-425^{Ile}. 24 water molecules that are in close proximity to FeMoco.

QM-region details for E_4/E_4-N_2/E_4-N_2' calculations (Figure S4):
FeMoco, the side chain of residues directly coordinating Femoco (α-442^{His}, α-275^{Cys}), the side chain of neighboring charged residues (α-96^{Arg}, α-359^{Arg}, α-380^{Glu}), the side chain of residues capable of participating in hydrogen bonding (α-195^{His}, α-191^{Gln}, α-278^{Ser}), as well as the side chain of spatially close residues (α-70^{Val}, α-381^{Phe}).
Total of 132 atoms for an E_0 redox state, 136 QM atoms in an E_4 redox state.

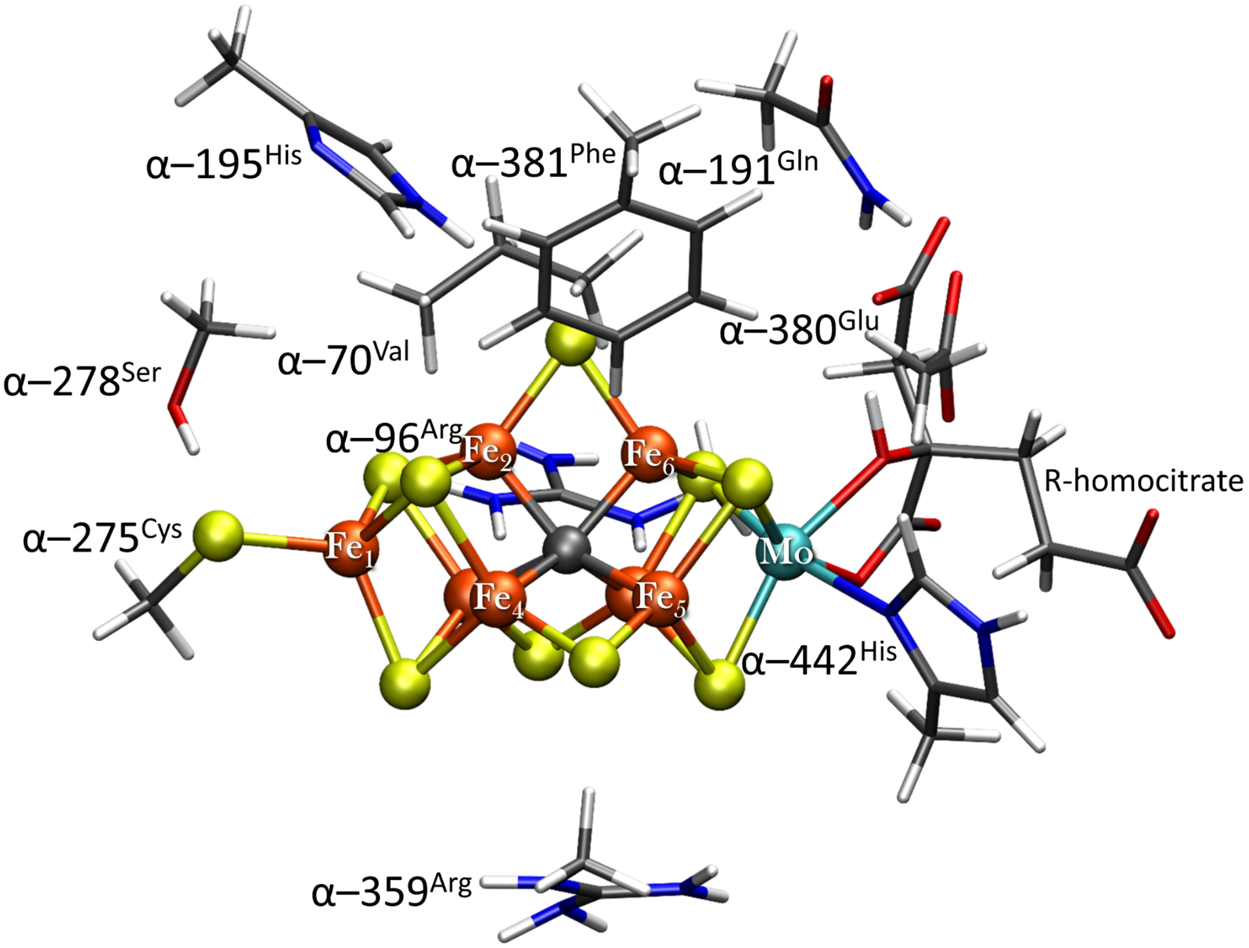

Figure S4. The QM-region used in the E_4/E_4-N_2/E_4-N_2' calculations. Fe. Link-atoms (shown in figure) are not counted. In E_4 models, 4 H atoms are added, giving a total QM-region of 136 atoms.

1. Benediktsson, B.; Bjornsson, R., QM/MM Study of the Nitrogenase MoFe Protein Resting State: Broken-Symmetry States, Protonation States, and QM Region Convergence in the FeMoco Active Site. *Inorganic Chemistry* **2017,** *56* (21), 13417-13429.
2. Abraham, M. J.; Murtola, T.; Schulz, R.; Páll, S.; Smith, J. C.; Hess, B.; Lindahl, E., GROMACS: High performance molecular simulations through multi-level parallelism from laptops to supercomputers. *SoftwareX* **2015,** *1-2*, 19-25.
3. Hess, B.; Kutzner, C.; van der Spoel, D.; Lindahl, E., GROMACS 4: Algorithms for Highly Efficient, Load-Balanced, and Scalable Molecular Simulation. *Journal of Chemical Theory and Computation* **2008,** *4* (3), 435-447.
4. Pronk, S.; Páll, S.; Schulz, R.; Larsson, P.; Bjelkmar, P.; Apostolov, R.; Shirts, M. R.; Smith, J. C.; Kasson, P. M.; van der Spoel, D.; Hess, B.; Lindahl, E., GROMACS 4.5: a high-throughput and highly parallel open source molecular simulation toolkit. *Bioinformatics* **2013,** *29* (7), 845-854.
5. Best, R. B.; Zhu, X.; Shim, J.; Lopes, P. E. M.; Mittal, J.; Feig, M.; MacKerell, A. D., Optimization of the Additive CHARMM All-Atom Protein Force Field Targeting Improved Sampling of the Backbone ϕ, ψ and Side-Chain χ1 and χ2 Dihedral Angles. *Journal of Chemical Theory and Computation* **2012,** *8* (9), 3257-3273.
6. Wright, L. B.; Rodger, P. M.; Walsh, T. R., Aqueous citrate: a first-principles and force-field molecular dynamics study. *RSC Advances* **2013,** *3* (37), 16399-16409.
7. Vanommeslaeghe, K.; Hatcher, E.; Acharya, C.; Kundu, S.; Zhong, S.; Shim, J.; Darian, E.; Guvench, O.; Lopes, P.; Vorobyov, I.; Mackerell Jr., A. D., CHARMM general force field: A force field for drug-like molecules compatible with the CHARMM all-atom additive biological force fields. *Journal of Computational Chemistry* **2010,** *31* (4), 671-690.
8. Spatzal, T.; Aksoyoglu, M.; Zhang, L.; Andrade, S. L. A.; Schleicher, E.; Weber, S.; Rees, D. C.; Einsle, O., Evidence for Interstitial Carbon in Nitrogenase FeMo Cofactor. *Science* **2011,** *334* (6058), 940-940.
9. Zhang, L.; Kaiser, J. T.; Meloni, G.; Yang, K.-Y.; Spatzal, T.; Andrade, S. L. A.; Einsle, O.; Howard, J. B.; Rees, D. C., The Sixteenth Iron in the Nitrogenase MoFe Protein. *Angewandte Chemie International Edition* **2013,** *52* (40), 10529-10532.
10. Li, H.; Robertson, A. D.; Jensen, J. H., Very fast empirical prediction and rationalization of protein pKa values. *Proteins: Structure, Function, and Bioinformatics* **2005,** *61* (4), 704-721.
11. Olsson, M. H. M.; Søndergaard, C. R.; Rostkowski, M.; Jensen, J. H., PROPKA3: Consistent Treatment of Internal and Surface Residues in Empirical pKa Predictions. *Journal of Chemical Theory and Computation* **2011,** *7* (2), 525-537.
12. Nosé, S., A unified formulation of the constant temperature molecular dynamics methods. *The Journal of Chemical Physics* **1984,** *81* (1), 511-519.
13. Nosé, S., A molecular dynamics method for simulations in the canonical ensemble. *Molecular Physics* **1984,** *52* (2), 255-268.
14. Hoover, W. G., Canonical dynamics: Equilibrium phase-space distributions. *Physical Review A* **1985,** *31* (3), 1695-1697.
15. Martyna, G. J.; Klein, M. L.; Tuckerman, M., Nosé–Hoover chains: The canonical ensemble via continuous dynamics. *The Journal of Chemical Physics* **1992,** *97* (4), 2635-2643.

E_4 models

References for E_4 models in Figure 2 of article:

a: L. Rao, X. Xu, C. Adamo, *ACS. Catal.* **2016**, *6*, 1567-1577.
c, f, g: L. Cao, O. Caldararu, U. Ryde, *J. Chem. Theory Comput.* **2018**, *14*, 6653-6678.
d: P. E. M. Siegbahn, *J. Am. Chem. Soc.* **2016**, *138*, 10485-10495.
h: I. Dance, *J. Am. Chem. Soc.* **2007**, *129*, 1076-1088.
e, m, n, o: S. Raugei, L. C. Seefeldt, B. M. Hoffman, *Proc. Natl. Acad. Sci.* **2018**, 115, E10521-E10530.
i: J. Vertemara, PhD thesis. Università degli Studi di Milano-Bicocca, **2018**.

Coordinates of E_4 models

Cartesian coordinates of the QM region for all QM/MM-optimized E_4/E_4-N_2/E_4-N_2' models are available as XYZ files in a compressed archive.

Table S1. Calculated relative TPSSh energies (both polarized QM and QM/MM energies are shown) and Mulliken spin populations on the metal ions for all the E_4 models (**a**-**o**) investigated in Fig. 2. Only the BS solutions with the lowest energy shown.

Model	QM	QM/MM	Mo	Fe_1	Fe_2	Fe_3	Fe_4	Fe_5	Fe_6	Fe_7
a)-147	31.46	23.67	-0.29	-3.44	2.69	3.18	-3.26	3.12	2.42	-2.81
b)-247	20.40	17.66	-0.48	3.49	-2.82	1.48	-3.23	3.02	2.63	-3.08
c)-247	17.14	14.73	-0.46	2.25	-2.77	2.89	-3.32	3.10	2.39	-2.90
d)-147	16.96	17.58	-0.51	-3.39	3.15	3.39	-2.93	2.57	1.92	-2.77
e)-147	16.88	11.23	-0.70	-3.46	2.69	3.02	-3.38	3.02	2.13	-1.42
f)-346	21.80	22.55	-0.86	3.41	0.13	-3.29	-3.32	2.30	-0.15	2.79
g)-346	17.60	13.02	-0.20	3.42	1.31	-3.26	-2.67	2.38	-2.91	2.84
h)-247	12.56	11.37	0.27	3.40	-2.51	3.29	-3.22	3.04	-0.88	-2.91
i)-247	12.55	10.91	-0.32	3.37	-3.14	3.06	-3.14	3.05	1.29	-2.98
j)-147	4.99	3.35	-0.51	-3.43	2.73	3.21	-3.28	3.00	2.13	-2.46
k)-147	3.29	1.95	-0.43	-3.42	2.70	3.13	-3.29	2.97	2.15	-2.35
l)-147	3.75	2.44	-0.49	-3.43	2.53	2.99	-3.30	3.07	2.59	-2.30
m)-346	2.47	2.04	-0.55	-3.40	2.53	3.13	-3.24	2.97	2.36	-2.43
n)-346	0.00	0.00	-0.32	3.35	2.22	-3.17	-3.29	3.02	-3.29	2.76
o)-147	2.48	2.25	-0.26	-3.44	3.00	3.14	-3.28	2.99	1.79	-2.41

Table S2. Calculated relative TPSSh energies (both polarized QM and QM/MM energies are shown) and Mulliken spin populations on the metal ions for all 4 broken-symmetry solutions (235, 346, 247, 147) of E_4 models (**a**-**o**) in Fig. 2.

Model	QM	QM/MM	Mo	Fe_1	Fe_2	Fe_3	Fe_4	Fe_5	Fe_6	Fe_7
a)-147	31.46	23.67	-0.29	-3.44	2.69	3.18	-3.26	3.12	2.42	-2.81
a)-235	35.63	29.53	0.43	3.44	-2.73	-3.17	3.37	-2.72	-1.92	3.06
a)-247	38.82	33.46	-0.07	3.36	-2.82	3.18	-3.18	3.08	0.35	-2.90
a)-346	41.63	35.97	-0.50	3.38	2.63	-3.48	-3.31	2.81	-2.68	2.74
b)-147	21.17	17.98	-0.56	-3.44	2.53	3.23	-3.31	3.01	2.95	-2.85
b)-235	31.05	27.53	-0.52	2.92	-3.33	-3.36	3.10	-3.12	2.95	2.85
b)-247	20.40	17.66	-0.48	3.49	-2.82	1.48	-3.23	3.02	2.63	-3.08
b)-346	25.35	22.19	-0.14	3.40	2.76	-3.44	-3.39	2.87	-3.08	2.65
c)-147	18.94	16.88	-0.57	-3.41	3.17	3.24	-3.57	3.25	2.45	-2.90
c)-235	24.85	21.89	-0.39	3.36	-3.46	-3.52	3.32	-3.19	2.38	2.91
c)-247	17.14	14.73	-0.46	2.25	-2.77	2.89	-3.32	3.10	2.39	-2.90
c)-346	28.79	22.93	-0.49	3.18	2.04	-3.60	-3.44	3.24	-2.60	2.92
d)-147	16.96	17.58	-0.51	-3.39	3.15	3.39	-2.93	2.57	1.92	-2.77
d)-235	19.91	18.28	-0.54	3.39	-3.26	-3.43	2.96	-2.60	1.83	2.69
d)-247	36.14	35.91	-0.48	3.13	-3.33	3.42	-2.86	2.48	1.71	-2.83
d)-346	21.84	24.07	-1.51	3.41	2.55	-3.36	-3.11	2.88	-3.15	3.04
e)-147	16.88	11.23	-0.70	-3.46	2.69	3.02	-3.38	3.02	2.13	-1.42
e)-235	23.10	17.47	-0.44	3.45	-3.28	-3.20	3.20	-3.16	1.41	2.87
e)-247	21.58	16.55	0.14	3.18	-3.12	3.18	-3.30	3.09	-2.08	-0.27
e)-346	24.51	18.95	-0.10	3.39	2.90	-3.36	-3.25	3.01	-1.65	0.04
f)-147	23.18	19.90	-0.10	-3.39	3.39	3.32	-2.90	2.62	0.74	-2.70
f)-235	22.87	20.17	-0.17	3.39	-3.14	-3.15	2.95	-2.73	0.85	2.87
f)-247	22.24	19.07	-0.08	3.38	-3.01	3.26	-3.11	2.34	-0.06	-2.01
f)-346	21.80	22.55	-0.86	3.41	0.13	-3.29	-3.32	2.30	-0.15	2.79

g)-147	22.24	16.35	-0.54	-3.39	2.37	3.24	-2.75	2.65	2.62	-2.88
g)-235	19.67	15.92	-0.26	3.36	-3.41	-3.27	2.67	-1.99	1.35	2.82
g)-247	30.14	24.57	-0.25	3.12	-3.34	3.08	-2.74	2.60	1.79	-2.83
g)-346	17.60	13.02	-0.20	3.42	1.31	-3.26	-2.67	2.38	-2.91	2.84
h)-147	14.55	14.36	-0.20	-3.41	2.74	3.26	-3.15	3.15	1.15	-2.39
h)-235	13.75	11.16	0.26	3.43	-2.24	-3.17	3.31	-3.08	-1.13	2.78
h)-247	12.56	11.37	0.27	3.40	-2.51	3.29	-3.22	3.04	-0.88	-2.91
h)-346	18.31	16.81	-0.72	3.45	-0.26	-3.34	-3.24	2.82	-0.51	2.55
i)-147	15.15	14.35	-0.44	-3.03	2.78	3.22	-3.19	3.08	1.88	-2.94
i)-235	12.72	12.83	-0.38	3.10	-3.13	-3.20	3.20	-3.12	1.74	2.89
i)-247	12.55	10.91	-0.32	3.37	-3.14	3.06	-3.14	3.05	1.29	-2.98
i)-346	19.64	16.34	-0.41	3.48	-0.44	-3.10	-3.08	3.19	-2.46	3.03
j)-147	4.99	3.35	-0.51	-3.43	2.73	3.21	-3.28	3.00	2.13	-2.46
j)-235	6.29	5.35	-0.47	3.39	-3.15	-3.23	3.29	-3.15	1.87	2.39
j)-247	14.30	13.43	-0.43	3.09	-3.48	3.10	-3.38	2.98	2.16	-2.58
j)-346	-18.16	-15.24	H_2 dissociates							
k)-147	3.29	1.95	-0.43	-3.42	2.70	3.13	-3.29	2.97	2.15	-2.35
k)-235	6.93	4.90	0.12	3.41	-2.79	-3.10	3.30	-3.08	0.70	2.21
k)-247	6.15	5.87	0.09	3.46	-2.79	3.18	-3.16	3.06	-0.71	-2.72
k)-346	11.90	11.05	-0.47	3.40	2.80	-3.28	-3.27	2.75	-2.64	2.13
l)-147	3.75	2.44	-0.49	-3.43	2.53	2.99	-3.30	3.07	2.59	-2.30
l)-235	6.68	5.73	-0.17	3.43	-2.80	-3.02	3.33	-3.04	0.35	2.48
l)-247	7.03	5.45	0.53	3.44	-2.41	3.28	-3.22	3.11	-2.23	-2.39
l)-346	12.04	11.68	-0.23	3.45	1.08	-3.19	-3.21	3.02	-2.86	2.76
m)-147	3.70	1.78	-0.55	-3.40	2.53	3.13	-3.24	2.97	2.36	-2.43
m)-235	7.29	4.61	0.17	3.40	-2.41	-2.91	3.25	-2.91	2.91	-1.30
m)-247	12.44	10.88	-0.43	3.15	-2.48	1.24	-3.18	3.02	2.65	-2.83
m)-346	2.47	2.04	-0.55	-3.40	2.53	3.13	-3.24	2.97	2.36	-2.43
n)-147	2.67	0.26	-0.52	-3.44	2.68	2.81	-3.34	3.04	2.96	-2.61
n)-235	5.03	4.13	-0.40	3.12	-3.07	-3.30	3.15	-3.20	2.73	2.44
n)-247	-5.75	-9.07	H_2 dissociates							
n)-346	0.00	0.00	-0.32	3.35	2.22	-3.17	-3.29	3.02	-3.29	2.76
o)-147	2.48	2.25	-0.26	-3.44	3.00	3.14	-3.28	2.99	1.79	-2.41
o)-235	5.36	5.90	-0.44	3.45	-3.04	-3.16	3.32	-3.03	1.06	2.47
o)-247	17.25	16.99	-0.51	3.18	-2.26	0.75	-3.19	2.98	2.84	-2.87
o)-346	6.08	4.78	-0.45	3.42	2.39	-3.11	-3.20	3.02	-3.28	2.44

Table S3. Calculated relative TPSS energies (polarized QM single-point energies on TPSSh geometries) and Mulliken spin populations on the metal ions for all 4 broken-symmetry solutions (235, 346, 247, 147) of E_4 models (**a**-**o**) in Fig. 2.

Model	QM	Mo	Fe_1	Fe_2	Fe_3	Fe_4	Fe_5	Fe_6	Fe_7
a)-147	38.90	-0.15	-3.18	2.33	2.82	-2.81	2.65	2.02	-2.29
a)-235	37.77	0.25	3.17	-1.77	-2.80	2.94	-2.61	-1.35	2.50
a)-247	39.25	-0.03	3.06	-2.42	2.76	-2.70	2.57	0.25	-2.49
a)-346	48.22	-0.20	3.10	2.19	-3.18	-2.84	2.16	-2.14	2.18
b)-147	25.99	-0.30	-3.15	2.29	2.82	-2.87	2.48	2.44	-2.38
b)-235	31.86	-0.24	2.19	-2.75	-2.78	2.64	-2.65	2.46	2.37
b)-247	15.77	-0.15	3.17	-2.11	0.88	-2.74	2.56	1.85	-2.58
b)-346	32.76	0.00	3.07	2.41	-3.00	-2.95	2.33	-2.65	2.02
c)-147	47.64	-0.30	-3.12	2.82	2.88	-3.31	2.99	2.05	-2.62
c)-235	51.28	-0.20	3.05	-3.07	-3.21	3.12	-2.94	1.73	2.59
c)-247	36.81	-0.27	2.02	-2.46	2.55	-3.01	2.81	2.00	-2.59
c)-346	48.97	-0.35	2.85	1.80	-3.27	-3.24	2.96	-2.25	2.54
d)-147	26.12	-0.26	-3.16	2.78	3.09	-2.52	2.22	1.58	-2.38
d)-235	28.94	-0.24	3.16	-2.88	-3.09	2.56	-2.18	1.17	2.26
d)-247	44.13	-0.04	3.20	-2.91	3.19	-2.39	2.12	0.06	-2.42

d)-346	40.53	-0.94	3.15	2.18	-3.07	-2.77	2.38	-2.78	2.67
e)-147	12.81	-0.36	-3.20	2.46	2.59	-3.12	2.50	0.61	0.29
e)-235	21.77	-0.05	3.21	-2.64	-2.57	2.96	-2.62	0.47	1.74
e)-247	18.02	0.02	2.87	-2.78	2.77	-2.94	2.59	-1.53	-0.02
e)-346	16.28	-0.10	3.13	2.17	-3.04	-2.93	2.42	0.07	-0.73
f)-147	15.44	-0.09	-3.10	2.98	2.81	-2.47	2.16	0.69	-1.90
f)-235	10.00	0.12	3.13	-2.52	-2.55	2.87	-2.19	-0.42	2.12
f)-247	8.15	-0.09	3.05	-2.55	2.82	-2.68	1.81	0.01	-1.48
f)-346	3.43	-0.38	3.09	-0.25	-2.77	-2.88	1.90	0.03	2.11
g)-147	12.32	-0.33	-3.10	1.81	2.76	-2.25	2.28	2.21	-2.21
g)-235	11.68	-0.16	3.07	-2.97	-2.74	2.18	-1.39	0.72	2.33
g)-247	13.60	0.05	3.18	-2.48	2.83	-2.19	1.44	0.21	-2.21
g)-346	4.07	-0.05	3.13	0.57	-2.73	-2.25	1.95	-2.24	2.30
h)-147	6.53	-0.18	-3.11	2.33	2.75	-2.68	2.69	0.95	-1.62
h)-235	4.91	0.13	3.12	-1.86	-2.69	2.96	-2.58	-0.73	2.07
h)-247	4.82	0.09	3.07	-2.08	2.84	-2.72	2.49	-0.58	-2.41
h)-346	2.87	-0.34	3.11	-0.44	-2.95	-2.73	2.30	-0.21	1.94
i)-147	14.67	-0.22	-2.64	2.49	2.80	-2.83	2.67	1.37	-2.44
i)-235	12.08	-0.07	3.01	-2.69	-2.69	2.83	-2.63	0.68	2.39
i)-247	14.50	-0.09	3.07	-2.69	2.63	-2.60	2.57	0.58	-2.48
i)-346	14.45	-0.26	3.18	-0.68	-2.64	-2.57	2.73	-1.94	2.54
j)-147	5.67	-0.30	-3.14	2.29	2.76	-2.88	2.60	1.63	-1.76
j)-235	3.50	0.07	3.10	-2.41	-2.56	2.97	-2.64	0.82	1.35
j)-247	10.65	0.01	3.20	-2.76	2.65	-2.78	2.59	-0.37	-1.74
j)-346	-3.41	-1.03	H_2 dissociates						
k)-147	1.97	-0.24	-3.13	2.27	2.67	-2.90	2.54	1.67	-1.63
k)-235	0.37	0.15	3.12	-2.29	-2.58	2.93	-2.59	0.49	1.49
k)-247	2.95	-0.03	3.16	-2.39	2.72	-2.74	2.56	-0.36	-2.19
k)-346	9.11	-0.10	3.14	2.41	-2.81	-2.76	2.15	-2.06	1.10
l)-147	3.41	-0.36	-3.14	2.16	2.50	-2.94	2.62	2.09	-1.51
l)-235	2.28	-0.03	3.13	-2.38	-2.53	2.96	-2.56	0.26	1.83
l)-247	4.46	0.18	3.13	-2.17	2.88	-2.81	2.62	-1.72	-1.65
l)-346	11.02	-0.10	3.11	0.89	-2.71	-2.74	2.52	-2.32	2.21
m)-147	1.58	-0.36	-3.10	1.99	2.63	-2.82	2.56	1.93	-1.69
m)-235	0.21	0.05	3.10	-1.89	-2.49	2.85	-2.54	2.40	-1.02
m)-247	2.77	-0.16	3.08	-1.78	-0.65	-2.65	2.58	2.13	-2.00
m)-346	12.52	-0.12	3.03	1.77	-2.65	-2.79	2.59	-2.99	2.34
n)-147	7.06	-0.31	-3.16	2.26	2.19	-2.97	2.67	2.50	-1.86
n)-235	7.88	0.00	2.61	-2.51	-2.73	2.86	-2.70	2.03	1.55
n)-247	-0.12	-0.12	H_2 dissociates						
n)-346	6.73	-0.13	3.07	1.84	-2.69	-2.85	2.55	-2.82	2.16
o)-147	0.00	-0.24	-3.15	2.59	2.56	-2.94	2.62	1.38	-1.35
o)-235	1.02	0.12	3.17	-2.23	-2.58	3.02	-2.46	-0.45	1.79
o)-247	5.41	-0.22	3.14	-1.61	-1.27	-2.64	2.53	2.34	-1.97
o)-346	5.51	-0.15	3.12	1.92	-2.53	-2.69	2.54	-2.81	1.65

Table S4. Calculated relative B3LYP energies (polarized QM single-point energies on TPSSh geometries) and Mulliken spin populations on the metal ions for all 4 broken-symmetry solutions (235, 346, 247, 147) of E_4 models (**a**-**o**) in Fig. 2.

Model	QM	Mo	Fe_1	Fe_2	Fe_3	Fe_4	Fe_5	Fe_6	Fe_7
a)-147	36.83	-0.38	-3.48	2.79	3.29	-3.39	3.28	2.60	-2.98
a)-235	41.63	0.63	3.49	-2.83	-3.28	3.48	-2.94	-2.26	3.23
a)-247	51.93	-0.01	3.41	-2.91	3.29	-3.32	3.26	0.19	-3.05
a)-346	45.17	-0.71	3.42	2.78	-3.52	-3.43	3.02	-2.83	2.97
b)-147	26.84	-0.68	-3.48	2.67	3.31	-3.41	3.17	3.11	-3.01
b)-235	35.93	-0.66	3.16	-3.49	-3.50	3.19	-3.28	3.13	3.03
b)-247	33.94	-0.65	3.55	-3.03	1.71	-3.37	3.15	2.91	-3.21
b)-346	27.71	-0.25	3.44	2.90	-3.53	-3.49	3.07	-3.22	2.88
c)-147	2.46	-0.69	-3.47	3.25	3.34	-3.60	3.32	2.67	-3.03
c)-235	11.06	-0.50	3.41	-3.53	-3.58	3.38	-3.27	2.60	3.06
c)-247	11.17	-0.56	2.17	-2.88	3.10	-3.39	3.21	2.55	-3.04
c)-346	18.34	-0.56	3.29	2.18	-3.68	-3.47	3.31	-2.83	3.07
d)-147	25.02	-0.62	-3.43	3.26	3.42	-3.10	2.75	2.18	-2.95
d)-235	26.50	-0.65	3.43	-3.38	-3.51	3.10	-2.82	2.22	2.87
d)-247	42.52	-0.56	3.28	-3.41	3.46	-3.02	2.65	1.95	-3.00
d)-346	15.70	-1.81	3.43	2.73	-3.42	-3.21	3.05	-3.23	3.16
e)-147	35.47	-0.81	-3.50	2.79	3.16	-3.45	3.16	2.34	-1.73
e)-235	34.70	-0.66	3.48	-3.39	-3.34	3.32	-3.30	1.87	3.02
e)-247	41.38	0.20	3.31	-3.24	3.29	-3.39	3.26	-2.40	-0.24
e)-346	45.13	-0.03	3.43	3.13	-3.41	-3.37	3.20	-2.09	0.04
f)-147	43.90	-0.28	-3.43	3.49	3.45	-3.06	2.81	0.96	-2.99
f)-235	43.19	-0.36	3.43	-3.27	-3.31	3.12	-2.94	1.19	3.08
f)-247	50.30	0.13	3.44	-3.16	3.39	-3.24	2.64	-0.17	-2.43
f)-346	53.73	-1.09	3.43	0.77	-3.44	-3.44	2.50	-0.53	3.07
g)-147	42.86	-0.73	-3.44	2.62	3.39	-2.99	2.68	2.95	-3.11
g)-235	38.21	-0.30	3.41	-3.51	-3.43	2.81	-2.32	1.74	3.03
g)-247	50.04	-0.43	3.25	-3.48	3.19	-2.83	2.79	2.15	-3.08
g)-346	40.00	-0.39	3.44	1.88	-3.42	-2.79	2.59	-3.19	3.05
h)-147	33.76	-0.26	-3.46	2.82	3.42	-3.29	3.30	1.39	-2.71
h)-235	35.63	0.42	3.48	-2.39	-3.28	3.41	-3.25	-1.49	3.05
h)-247	33.04	0.54	3.45	-2.64	3.42	-3.36	3.21	-1.23	-3.08
h)-346	48.32	-0.62	3.40	0.66	-3.49	-3.42	3.04	-1.31	2.88
i)-147	29.01	-0.58	-3.20	2.90	3.35	-3.26	3.19	2.18	-3.13
i)-235	23.37	-0.54	3.26	-3.28	-3.34	3.26	-3.29	2.10	3.05
i)-247	19.60	-0.51	3.42	-3.31	3.19	-3.32	3.18	1.87	-3.18
i)-346	37.50	-0.42	3.48	0.03	-3.27	-3.25	3.33	-2.76	3.21
j)-147	12.34	-0.57	-3.47	2.88	3.29	-3.39	3.12	2.40	-2.75
j)-235	12.59	-0.79	3.44	-3.34	-3.37	3.40	-3.29	2.26	2.71
j)-247	17.60	-0.52	3.24	-3.61	3.20	-3.49	3.12	2.48	-2.85
j)-346	-24.11	H_2 dissociates							
k)-147	13.18	-0.51	-3.46	2.84	3.25	-3.40	3.13	2.40	-2.67
k)-235	23.14	0.05	3.46	-2.93	-3.24	3.39	-3.22	0.77	2.48
k)-247	19.58	0.42	3.51	-2.92	3.32	-3.25	3.23	-1.24	-2.93
k)-346	21.12	-0.67	3.44	2.96	-3.36	-3.40	2.97	-2.89	2.54
l)-147	14.52	-0.48	-3.47	2.67	3.15	-3.40	3.22	2.77	-2.69

l)-235	22.48	-0.19	3.48	-2.91	-3.15	3.42	-3.19	0.13	2.78
l)-247	20.04	0.72	3.49	-2.54	3.40	-3.28	3.27	-2.51	-2.69
l)-346	24.25	-0.30	3.52	1.18	-3.30	-3.33	3.19	-3.06	2.97
m)-147	11.78	-0.62	-3.45	2.69	3.25	-3.37	3.12	2.58	-2.72
m)-235	22.60	0.40	3.45	-2.53	-2.99	3.38	-3.03	3.19	-1.93
m)-247	25.87	-0.53	3.27	-2.63	1.39	-3.28	3.17	2.85	-3.03
m)-346	0.00	-1.85	3.44	2.98	-3.28	-3.36	3.27	-3.34	3.12
n)-147	5.56	-0.66	-3.47	2.89	3.01	-3.44	3.14	3.19	-2.93
n)-235	5.55	-0.67	3.28	-3.21	-3.43	3.22	-3.33	3.00	2.74
n)-247	-6.56	H_2 dissociates							
n)-346	1.40	-0.43	3.41	2.34	-3.29	-3.40	3.18	-3.43	2.98
o)-147	13.11	-0.35	-3.47	3.11	3.26	-3.40	3.15	2.02	-2.71
o)-235	16.53	-0.62	3.49	-3.21	-3.25	3.42	-3.20	1.36	2.75
o)-247	33.50	-0.68	3.29	-2.53	1.12	-3.31	3.16	3.08	-3.10
o)-346	12.96	-0.57	3.46	2.58	-3.25	-3.33	3.13	-3.41	2.68

Table S5. Calculated relative M06-2X energies (polarized QM single-point energies on TPSSh geometries) and Mulliken spin populations on the metal ions for all 4 broken-symmetry solutions (235, 346, 247, 147) of E_4 models (**a**-**o**) in Fig. 2.

Model	QM	Mo	Fe_1	Fe_2	Fe_3	Fe_4	Fe_5	Fe_6	Fe_7
a)-147	59.26	-0.68	-3.63	3.15	3.50	-3.77	3.65	3.04	-3.34
a)-235	63.14	1.00	3.63	-3.16	-3.50	3.83	-3.36	-2.92	3.60
a)-247	100.83	-0.57	3.59	-3.19	3.54	-3.70	3.65	0.79	-3.30
a)-346	63.66	-0.90	3.58	3.22	-3.62	-3.79	3.34	-3.34	3.33
b)-147	60.41	-0.85	-3.62	3.06	3.46	-3.77	3.49	3.52	-3.61
b)-235	57.97	-0.91	3.53	-3.84	-3.84	3.46	-3.71	3.60	3.41
b)-247	88.34	-0.95	3.88	-3.32	1.88	-3.67	3.38	3.50	-3.63
b)-346	47.33	-0.61	3.61	3.28	-3.80	-3.75	3.51	-3.57	3.33
c)-147	0.00	-0.83	-3.59	3.46	3.53	-3.85	3.56	3.16	-3.33
c)-235	2.06	-0.74	3.59	-3.61	-3.87	3.60	-3.54	3.07	3.35
c)-247	40.09	-0.77	1.93	-3.20	3.46	-3.55	3.46	3.01	-3.34
c)-346	28.84	-0.78	3.54	3.09	-3.97	-3.63	3.54	-3.33	3.36
d)-147	41.68	-0.81	-3.59	3.38	3.51	-3.44	3.23	3.02	-3.25
d)-235	43.28	-0.80	3.60	-3.53	-3.83	3.38	-3.32	3.09	3.26
d)-247	55.06	-0.83	3.58	-3.75	3.53	-3.41	3.25	2.99	-3.27
d)-346	12.25	-2.49	3.58	3.12	-3.55	-3.43	3.39	-3.43	3.41
e)-147	96.33	-0.84	-3.63	3.33	3.43	-3.80	3.49	2.35	-2.62
e)-235	61.35	-1.01	3.62	-3.51	-3.70	3.74	-3.60	2.73	3.30
e)-247	96.43	0.78	3.55	-3.47	3.55	-3.73	3.59	-3.06	-0.78
e)-346	107.91	0.01	3.59	3.49	-3.55	-3.68	3.57	-2.72	-0.25
f)-147	121.03	-0.68	-3.60	3.77	3.75	-3.42	3.35	1.46	-3.51
f)-235	114.39	-0.88	3.59	-3.43	-3.69	3.44	-3.42	2.13	3.49
f)-247	146.92	1.09	3.59	-3.41	3.79	-3.53	3.17	-1.18	-3.08
f)-346	157.39	-2.18	3.56	1.74	-3.75	-3.78	2.92	-0.97	3.55
g)-147	104.64	-0.95	-3.59	3.11	3.72	-3.64	3.00	3.31	-3.53
g)-235	113.17	-0.72	3.59	-3.72	-3.74	3.20	-2.88	2.54	3.50
g)-247	118.43	-0.71	3.52	-3.75	3.46	-3.08	3.40	2.77	-3.53
g)-346	113.17	-0.73	3.56	2.79	-3.74	-3.11	3.00	-3.67	3.49
h)-147	99.37	-0.69	-3.61	3.10	3.78	-3.68	3.65	2.30	-3.23

h)-235	105.60	0.79	3.62	-2.94	-3.43	3.77	-3.61	-2.46	3.56
h)-247	100.05	0.99	3.59	-2.98	3.82	-3.68	3.59	-2.40	-3.29
h)-346	141.35	-0.70	3.92	-1.09	-3.46	-3.56	3.33	-2.47	3.37
i)-147	74.64	-0.93	-3.53	3.18	3.65	-3.46	3.40	2.98	-3.55
i)-235	62.95	-0.93	3.57	-3.46	-3.63	3.50	-3.65	2.95	3.36
i)-247	58.24	-0.89	3.60	-3.46	3.48	-3.67	3.44	2.79	-3.59
i)-346	97.21	-0.66	3.59	0.50	-3.55	-3.49	3.67	-3.32	3.59
j)-147	41.25	-0.80	-3.61	3.29	3.44	-3.78	3.33	3.19	-3.33
j)-235	45.16	-1.31	3.60	-3.61	-3.55	3.80	-3.54	2.62	3.18
j)-247	31.41	-0.79	3.53	-3.90	3.50	-3.79	3.34	3.16	-3.28
j)-346	-35.61	-2.50	H_2 dissociates						
k)-147	57.42	-0.76	-3.60	3.15	3.45	-3.77	3.42	3.04	-3.26
k)-235	88.19	-1.00	3.60	-3.24	-3.45	3.79	-3.56	1.50	3.22
k)-247	72.63	0.88	3.60	-3.17	3.76	-3.41	3.61	-2.54	-3.33
k)-346	57.95	-0.86	3.59	3.38	-3.51	-3.74	3.30	-3.51	3.12
l)-147	55.05	-0.69	-3.61	3.03	3.45	-3.75	3.62	3.17	-3.25
l)-235	86.15	-0.92	3.61	-3.17	-3.42	3.78	-3.57	0.79	3.27
l)-247	64.81	0.99	3.59	-2.99	3.76	-3.45	3.63	-3.07	-3.20
l)-346	77.42	-0.62	3.89	0.91	-3.50	-3.55	3.56	-3.42	3.29
m)-147	54.25	-0.82	-3.60	3.16	3.47	-3.73	3.38	3.08	-3.30
m)-235	75.73	0.77	3.59	-2.76	-3.43	3.71	-3.31	3.73	-2.95
m)-247	87.20	-0.79	3.52	-3.19	1.74	-3.51	3.60	3.32	-3.41
m)-346	10.29	-2.51	3.59	3.56	-3.47	-3.68	3.69	-3.56	3.41
n)-147	18.54	-0.87	-3.61	3.31	3.38	-3.80	3.36	3.36	-3.45
n)-235	13.89	-1.06	3.54	-3.35	-3.73	3.50	-3.63	3.37	3.21
n)-247	-0.61	-1.01	H_2 dissociates						
n)-346	26.54	-0.70	3.59	2.60	-3.47	-3.73	3.51	-3.78	3.37
o)-147	57.22	-0.73	-3.61	3.49	3.49	-3.77	3.40	2.73	-3.22
o)-235	60.66	-1.10	3.61	-3.55	-3.44	3.80	-3.54	2.33	3.22
o)-247	95.07	-1.04	3.52	-3.26	1.69	-3.44	3.56	3.40	-3.54
o)-346	44.50	-0.83	3.59	3.40	-3.45	-3.69	3.37	-3.73	3.22

Table S6. Calculated relative TPSSh energies for different BS solutions (both polarized QM and QM/MM energies are shown) for **E_4-l** and **E_4-o** models, N_2-bound models (**E_4-l-N_2** and **E_4-o-N_2**) and models after reductive elimination (**E_4-l-N_2'** and **E_4-o-N_2'**).

Model	QM	QM/MM	Mo	Fe_1	Fe_2	Fe_3	Fe_4	Fe_5	Fe_6	Fe_7
l)-147	1.27	0.20	-0.49	-3.43	2.53	2.99	-3.30	3.07	2.59	-2.30
l)-235	4.20	3.48	-0.17	3.43	-2.80	-3.02	3.33	-3.04	0.35	2.48
l)-247	4.55	3.20	0.53	3.44	-2.41	3.28	-3.22	3.11	-2.23	-2.39
l)-346	9.56	9.43	-0.23	3.45	1.08	-3.19	-3.21	3.02	-2.86	2.76
o)-147	0.00	0.00	-0.26	-3.44	3.00	3.14	-3.28	2.99	1.79	-2.41
o)-235	2.88	3.65	-0.44	3.45	-3.04	-3.16	3.32	-3.03	1.06	2.47
o)-247	14.77	14.74	-0.51	3.18	-2.26	0.75	-3.19	2.98	2.84	-2.87
o)-346	3.60	2.54	-0.45	3.42	2.39	-3.11	-3.20	3.02	-3.28	2.44
l)-N2-147	4.42	4.54	-0.15	-3.39	2.63	3.21	-3.01	3.23	0.78	-2.27
l)-N2-235	0.00	0.00	0.08	3.43	-2.62	-2.87	3.29	-3.08	-0.46	2.58
l)-N2-247	2.81	2.93	0.29	3.42	-2.66	3.03	-3.18	3.03	-0.74	-2.70

l)-N2-346	14.27	12.55	-0.97	3.42	0.53	-3.28	-3.23	2.89	0.42	1.44
o)-N2-147	10.12	9.56	-0.59	-3.36	1.40	3.24	-3.30	3.07	3.06	-2.15
o)-N2-235	6.02	4.32	0.46	3.39	-1.52	-2.97	3.35	-3.00	-2.07	2.46
o)-N2-247	8.62	7.90	0.58	3.40	-1.49	3.16	-3.20	3.00	-2.49	-2.60
o)-N2-346	3.31	4.02	-0.17	3.45	0.18	-3.12	-3.18	3.02	-2.42	2.86
l)-N2'-147	1.81	0.81	-0.55	-3.39	3.33	3.20	-3.30	2.95	1.64	-2.51
l)-N2'-235	0.00	0.00	-0.41	3.38	-3.20	-3.25	3.24	-3.12	1.92	2.56
l)-N2'-247	5.64	5.84	-0.35	3.40	-3.26	2.97	-3.21	2.93	1.63	-2.91
l)-N2'-346	7.57	7.29	-0.29	3.20	3.20	-3.30	-3.34	2.97	-2.19	0.88
o)-N2'-147	4.41	1.15	-0.55	-3.42	2.10	3.10	-3.32	3.04	3.00	-2.37
o)-N2'-235	N_2 unbinds									
o)-N2'-247	N_2 unbinds									
o)-N2'-346	0.12	-1.74	-0.22	3.38	2.23	-3.24	-3.30	3.00	-3.09	2.80

Table S7. τ_4 parameters for all Fe ions in E_0, E_4, E_4-N_2 and E_4-N_2' models. $\tau_4 = \frac{360°-(\alpha+\beta)}{360°-2\theta}$ where α and β are the two greatest valence angles of the iron and θ is the tetrahedral angle, 109.5°, as proposed by Yang, et al. *Dalton Transactions* **2007**, 9, 955-964. An ideal tetrahedral geometry would have α=β=109.5° and τ_4=1, while ideal square-planar geometry would have α=β=180° and τ_4=0 and an ideal seesaw geometry would have α=120, β=180 τ_4≈0.43. In the case of E_4 and E_4-N_2, as reductive elimination removes hydrides coordinating Fe_2 and Fe_6 resulting in distorted tetrahedral geometry, pseudo τ_4 parameters (coordination of the hydrides is ignored) were calculated giving insight into how much the local geometry changes before and after reductive elimination.

	Fe_1	Fe_2	Fe_3	Fe_4	Fe_5	Fe_6	Fe_7
**E_0-235**	0.857	0.847	0.848	0.857	0.841	0.882	0.894
**E_4-l-147**	0.838	0.636*	0.854	0.856	0.847	NA	0.895
**E_4-o-147**	0.845	NA	0.867	0.848	0.833	0.649*	0.901
**E_4-l-N_2-235**	0.837	0.669*	0.877	0.866	0.853	0.645*	0.892
**E_4-o-N_2-346**	0.845	0.586*	0.886	0.861	0.857	0.673*	0.883
**E_4-l-N_2'-235**	0.820	0.766	0.886	0.858	0.853	0.749	0.894
**E_4-o-N_2'-346**	0.828	0.650	0.890	0.864	0.870	0.825	0.863

* indicates a pseudo τ_4 where coordination of the hydrides is ignored.

Table S8. τ_4' parameter for all Fe ions in E_0, E_4, E_4-N_2 and E_4-N_2' models. $\tau_{4'} = \frac{\beta-\alpha}{360°-\theta} + \frac{180°-\beta}{180°-\theta}$ where β >α, α and β are the two greatest valence angles of the iron and θ is the tetrahedral angle, 109.5°, and this formula does distinguish between α and β angles as proposed by Okuniewski, et al., *Polyhedron **2015,** 90, 47-57.* An ideal tetrahedral geometry would have α=β=109.5° and τ_4'=1, while an ideal square-planar geometry would have α=β=180° τ_4'=0 and an ideal seesaw geometry would have α=120, β=180 τ_4'≈0.24. In the case of E_4 and E_4-N_2, as reductive elimination removes hydrides coordinating Fe_2 and Fe_6 resulting in distorted tetrahedral geometry, pseudo τ_4 parameters (coordination of the hydrides is ignored) were calculated giving insight into how much the local geometry changes before and after reductive elimination.

	Fe_1	Fe_2	Fe_3	Fe_4	Fe_5	Fe_6	Fe_7
**E_0-235**	0.844	0.838	0.834	0.851	0.822	0.876	0.881
**E_4-l-147**	0.834	0.462*	0.835	0.831	0.839	NA	0.861
**E_4-o-147**	0.824	NA	0.864	0.845	0.825	0.487*	0.869
**E_4-l-N_2-235**	0.827	0.508*	0.861	0.844	0.844	0.489*	0.859
**E_4-o-N_2-346**	0.817	0.389*	0.879	0.845	0.857	0.494*	0.854
**E_4-l-N_2'-235**	0.800	0.641	0.897	0.854	0.843	0.641	0.871
**E_4-o-N_2'-346**	0.811	0.491	0.844	0.859	0.835	0.739	0.825

* indicates a pseudo τ_4' where coordination of the hydrides is ignored.

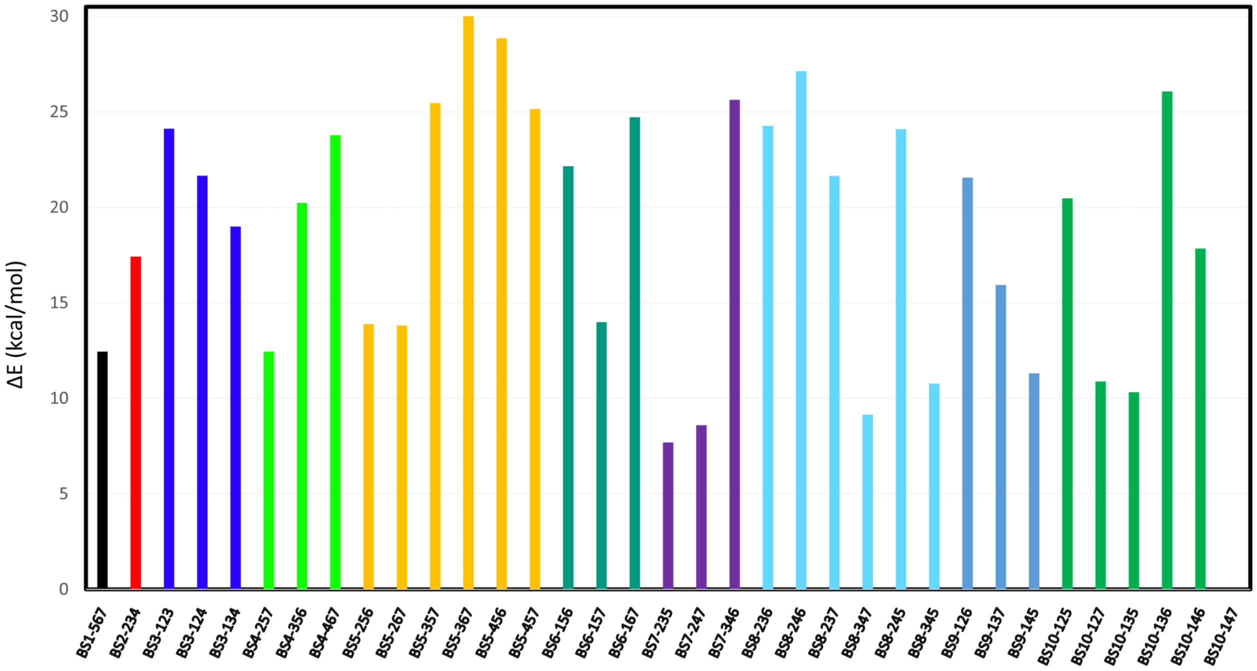

Figure S5. All 35 BS solutions for the **E_4-l** model (optimized geometry with BS-147 solution).

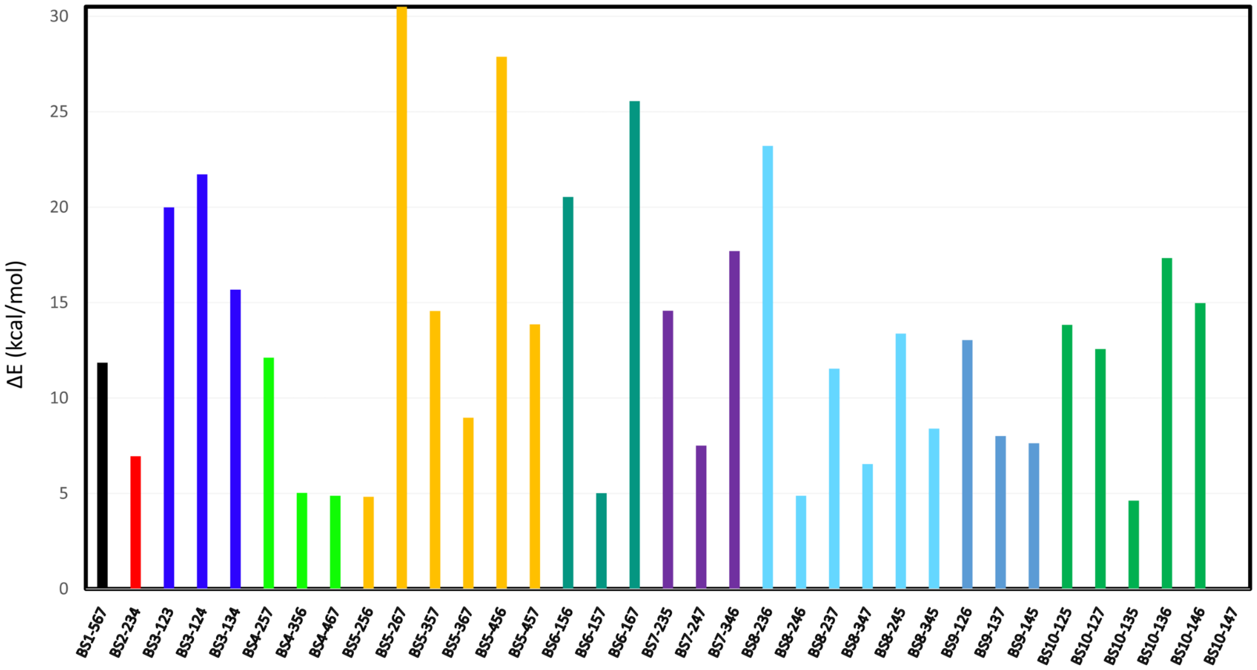

Figure S6. All 35 BS solutions for the **E₄-o** model (optimized geometry with BS-147 solution). BS-267 is 38 kcal/mol higher in energy than BS-147.

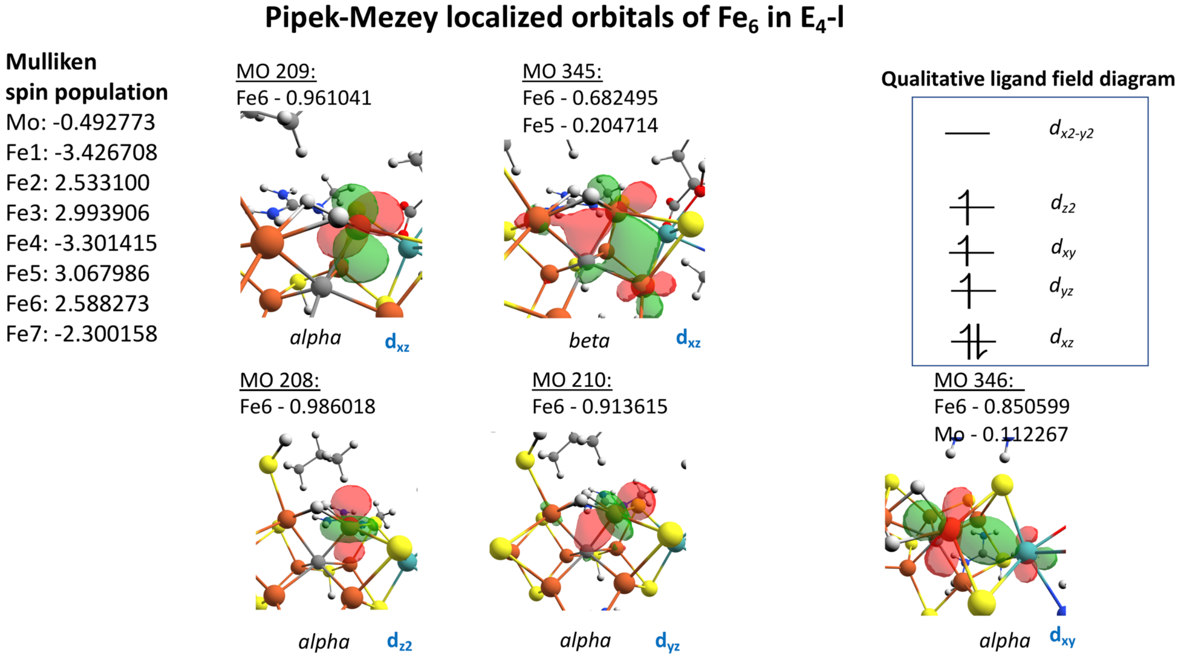

Figure S7. Pipek-Mezey localized orbitals associated with Fe_6 in the **E₄-I** model (BS-147 solution). A qualitative ligand-field diagram is shown based on the interpretation of the localized orbitals, suggesting an S=3/2 Fe(III) ion. Mulliken atomic spin populations of this state are also shown.

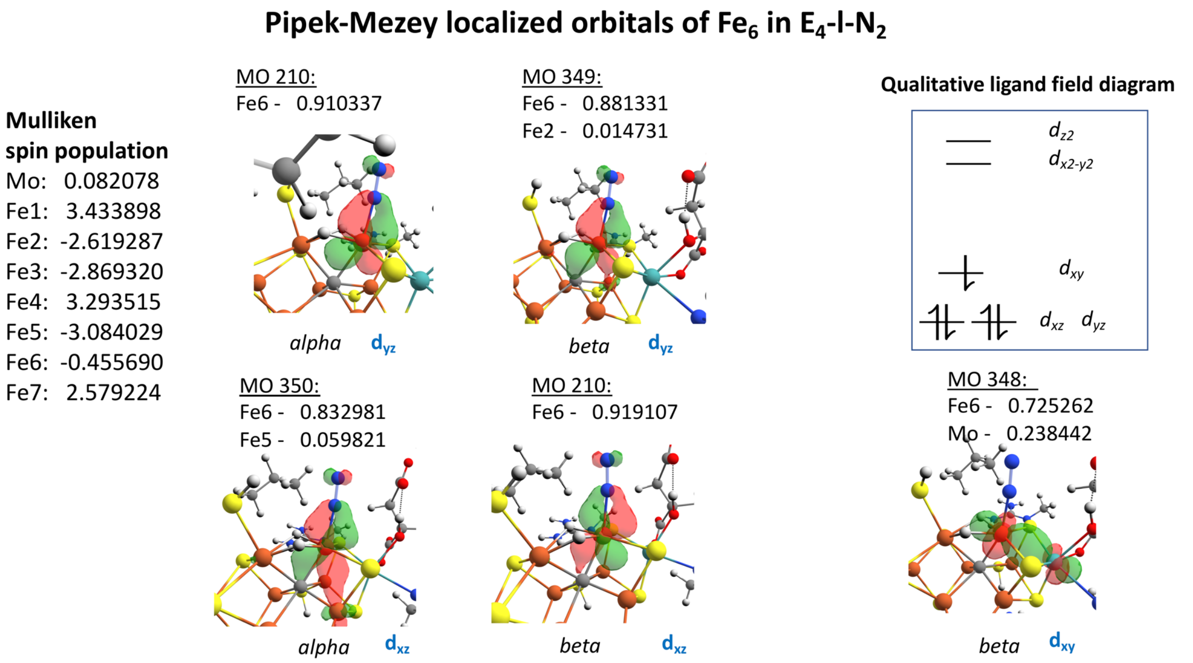

Figure S8. Pipek-Mezey localized orbitals of Fe_6 in **E_4-I-N_2** model (BS-235 solution). A qualitative ligand-field diagram is shown based on the interpretation of the localized orbitals which suggests Fe_6 to be a low-spin S=1/2 Fe(III). The increased population of backbonding d_{xz} and d_{yz} orbitals should aid dinitrogen binding. Mulliken atomic spin populations of this state are also shown.

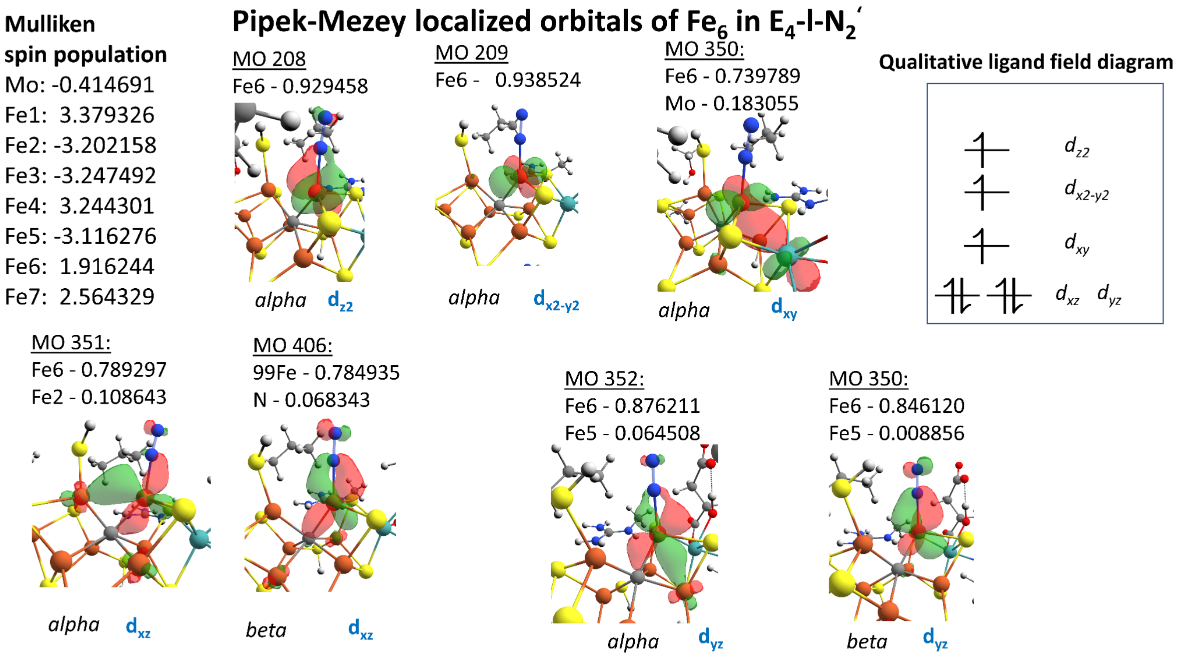

Figure S9. Pipek-Mezey localized orbitals of Fe_6 in **E_4-I-N_2**' model (BS-235 solution). A qualitative ligand-field diagram is shown based on the interpretation of the localized orbitals which suggests Fe_6 to be an S=3/2 Fe(I). The increased population of backbonding d_{xz} and d_{yz} orbitals should aid dinitrogen binding. Mulliken atomic spin populations of this state are also shown.

E_0 geometric comparison

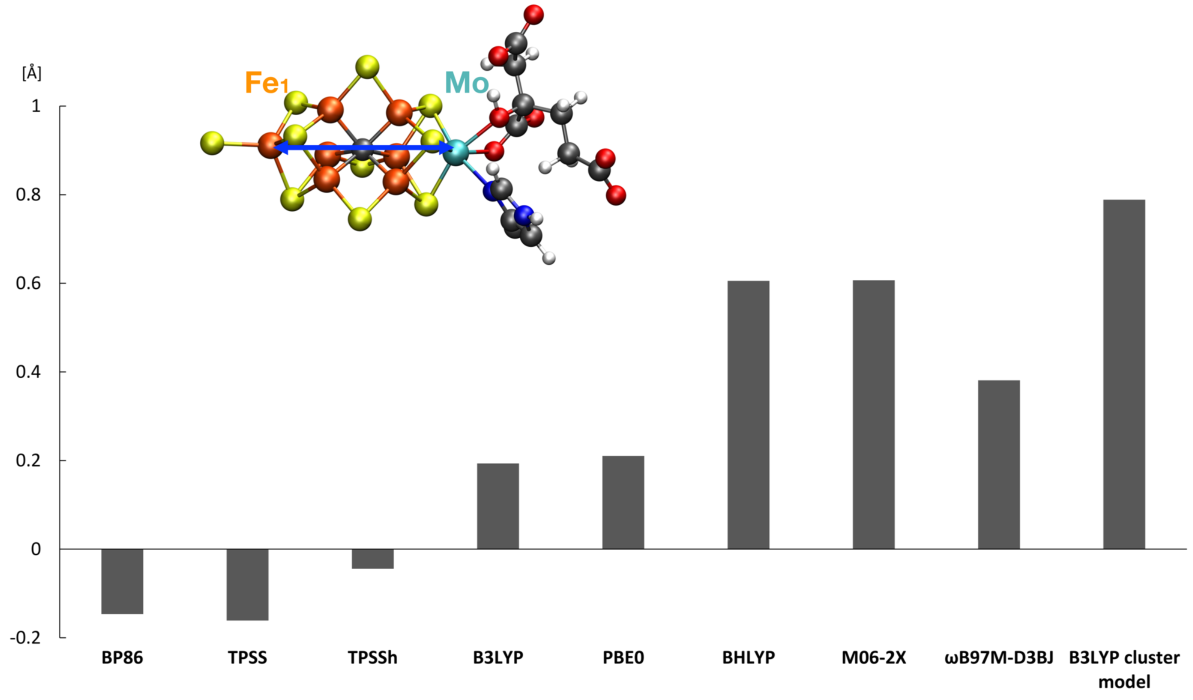

Figure S10. Deviation with respect to X-ray crystal structure (PDB ID : 3U7Q) for the Mo-Fe1 distance for various functionals calculated with QM/MM models (54 atom QM regions) or cluster models. B3LYP cluster model geometry is from Siegbahn (P. E. M. Siegbahn, *Phys. Chem. Chem. Phys.* **2019**, *21*, 15747-15759.)

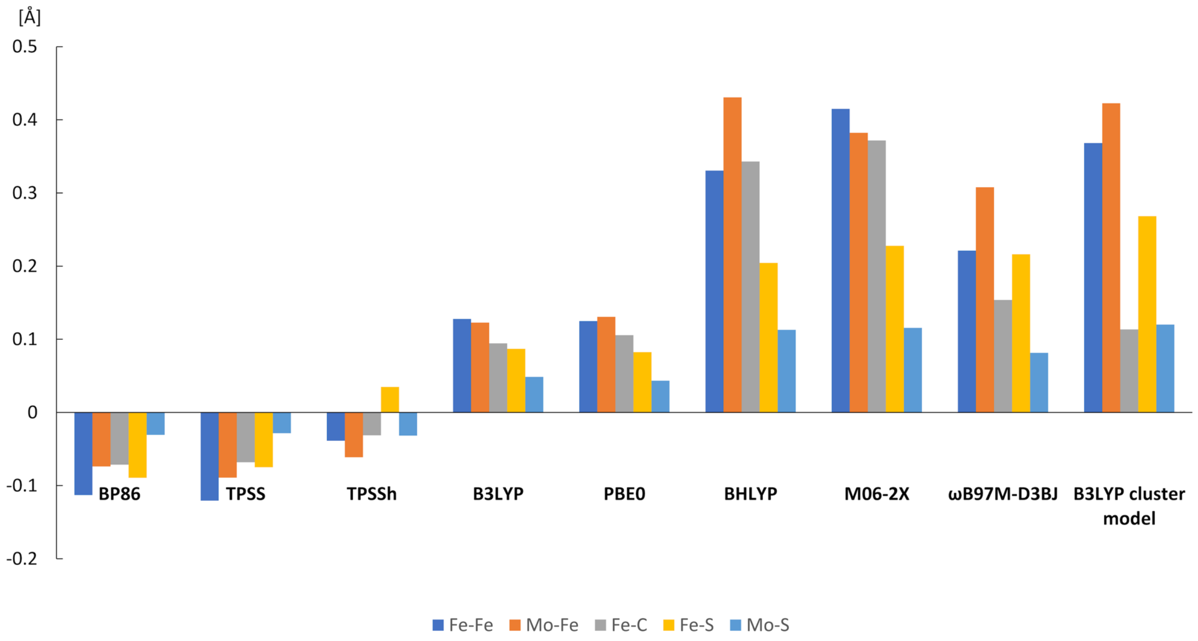

Figure S11. The largest deviation w.r.t. crystal structure for Fe-Fe, Mo-Fe, Fe-C, Fe-S and Mo-S distances for various QM/MM models (54 atom QM regions) with different functionals or a B3LYP cluster model. B3LYP cluster model geometry is from Siegbahn (P. E. M. Siegbahn, *Phys. Chem. Chem. Phys.* **2019**, *21*, 15747-15759.)

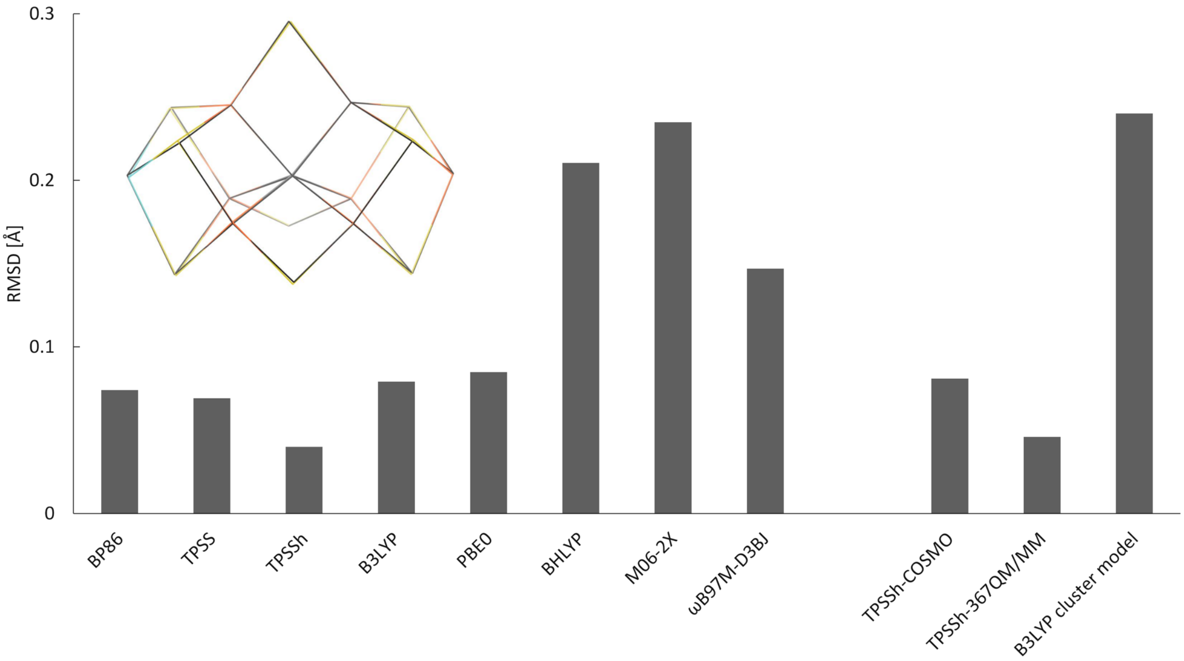

Figure S12. Root mean square deviations of the [$MoFe_7S_9C$] cluster w.r.t. crystal structure for various QM/MM models (54 atom QM regions) with different functionals, a TPSSh cluster model (54 atoms), a large QM-region (367 atoms) TPSSh-QM/MM model and a B3LYP cluster model. B3LYP cluster model geometry is from Siegbahn (P. E. M. Siegbahn, *Phys. Chem. Chem. Phys.* **2019**, *21*, 15747-15759.). Also shown is a superimposition of the TPSSh-QM/MM geometry (colored) and the X-ray crystal structure geometry (black).

Electronic structure of the resting state E_0 with different functionals

Tables S9-S11 show Mulliken spin populations on the FeMoco cluster atoms with various functionals, demonstrating a large change in electronic structure, whether using the X-ray geometry (Table S9), TPSSh-QM/MM geometry (Table S10), or an optimized geometry at each calculation level (Table S11). Figure S13 shows the relationship between atom spin populations (Mo,Fe,S,C) and various distances in the cofactor demonstrating e.g. how an increased Mo spin population correlates with long Mo-Fe distance. The latter relationship is also shown in Figure S14 with spin density isosurfaces shown as well.

Table S9. Mulliken spin populations of E_0 with different functionals calculated on the X-ray crystal structure geometry.

	BP86	TPSS	TPSSh	B3LYP	PBE0	ωB97M-D3BJ	BHLYP	M06-2X
Mo	-0.22	-0.25	-0.56	-0.73	-0.85	-0.93	-2.24	-0.16
Fe1	3.05	3.16	3.45	3.50	3.62	3.59	3.70	3.63
Fe2	-2.51	-2.70	-3.22	-3.35	-3.52	-3.50	-3.84	-3.84
Fe3	-2.55	-2.73	-3.23	-3.36	-3.53	-3.50	-3.66	-3.85
Fe4	2.84	2.97	3.33	3.43	3.60	3.66	3.94	3.85
Fe5	-2.52	-2.65	-3.15	-3.31	-3.50	-3.50	-3.65	-3.62
Fe6	2.19	2.35	2.95	3.14	3.34	3.34	3.87	3.33
Fe7	2.21	2.38	2.96	3.16	3.36	3.37	3.87	3.65
S1	0.15	0.14	0.16	0.18	0.18	0.17	0.12	0.09
S2	-0.08	-0.08	-0.10	-0.13	-0.14	-0.15	-0.19	-0.24
S3	0.13	0.13	0.14	0.16	0.16	0.16	0.17	0.07
S4	-0.01	0.00	0.00	-0.01	-0.02	-0.03	0.00	-0.07
S5	0.13	0.12	0.12	0.14	0.14	0.15	0.28	0.26
S6	-0.01	0.00	-0.01	-0.01	-0.02	-0.03	0.01	0.09
S7	0.03	0.02	0.01	0.01	0.02	0.02	0.07	-0.17
S8	-0.01	-0.01	0.00	0.00	0.00	0.02	0.15	0.11
S9	0.01	-0.01	-0.01	-0.01	0.00	0.02	0.14	-0.08
C	0.02	0.02	0.01	0.01	0.01	0.02	0.17	-0.15

Table S10. Mulliken spin populations of E_0 with different functionals calculated on the TPSSh-QM/MM geometry.

	BP86	TPSS	TPSSh	B3LYP	PBE0	ωB97M-D3BJ	BHLYP	M06-2X
Mo	-0.23	-0.25	-0.55	-0.72	-0.86	-0.99	-1.10	-1.05
Fe1	3.04	3.15	3.44	3.49	3.61	3.58	3.69	3.63
Fe2	-2.47	-2.67	-3.20	-3.35	-3.53	-3.55	-3.89	-3.75
Fe3	-2.59	-2.76	-3.24	-3.36	-3.52	-3.46	-3.62	-3.50
Fe4	2.82	2.94	3.30	3.40	3.57	3.62	3.88	3.81
Fe5	-2.53	-2.66	-3.12	-3.28	-3.47	-3.44	-3.73	-3.65
Fe6	2.23	2.40	2.97	3.17	3.36	3.36	3.58	3.43
Fe7	2.23	2.38	2.92	3.11	3.31	3.33	3.69	3.56
S1	0.15	0.14	0.16	0.17	0.17	0.16	0.12	0.12
S2	-0.06	-0.06	-0.09	-0.11	-0.13	-0.14	-0.17	-0.17
S3	0.12	0.12	0.14	0.16	0.17	0.18	0.21	0.20
S4	-0.02	-0.01	-0.02	-0.02	-0.03	-0.03	-0.08	-0.06
S5	0.15	0.13	0.13	0.15	0.15	0.14	0.14	0.18
S6	-0.01	0.00	0.00	-0.01	-0.01	-0.01	-0.02	0.00
S7	0.00	-0.01	-0.02	-0.02	-0.01	-0.03	-0.10	-0.10
S8	0.00	0.00	0.01	0.02	0.02	0.04	0.05	0.03
S9	0.01	0.00	0.00	0.01	0.03	0.05	0.12	0.12
C	0.03	0.04	0.04	0.04	0.05	0.07	0.15	0.11

Table S11. Mulliken spin populations of E_0 with different functionals calculated on the QM/MM optimized geometry for each respective functional. Also shown is a single-point B3LYP/LANL2DZ/6-31G* calculation (approx. same basis set as used by Siegbahn) on the B3LYP cluster model geometry from Siegbahn.

	BP86	TPSS	TPSSh	B3LYP	PBE0	ωB97M-D3BJ	BHLYP	M06-2X	B3LYP cluster model
Mo	-0.14	-0.16	-0.55	-0.95	-1.11	-1.74	-1.84	-1.90	-1.48
Fe1	2.80	2.96	3.44	3.55	3.65	3.62	3.76	3.72	3.60
Fe2	-2.05	-2.33	-3.20	-3.48	-3.66	-3.63	-4.08	-3.96	-3.53
Fe3	-2.20	-2.45	-3.24	-3.45	-3.58	-3.54	-3.76	-3.68	3.62
Fe4	2.65	2.81	3.30	3.51	3.67	3.78	4.06	4.00	-3.47
Fe5	-2.31	-2.47	-3.12	-3.36	-3.51	-3.50	-3.70	-3.66	3.42
Fe6	1.84	2.06	2.97	3.33	3.49	3.58	3.75	3.65	3.46
Fe7	1.95	2.13	2.92	3.26	3.44	3.50	3.99	3.88	-3.40
S1	0.12	0.13	0.16	0.17	0.16	0.17	0.10	0.12	-0.17
S2	-0.07	-0.08	-0.09	-0.13	-0.15	-0.11	-0.16	-0.16	0.16
S3	0.10	0.10	0.14	0.17	0.18	0.21	0.17	0.19	0.16
S4	-0.02	-0.02	-0.02	-0.02	-0.02	0.05	0.00	0.01	0.19
S5	0.15	0.14	0.13	0.17	0.17	0.17	0.18	0.22	0.05
S6	-0.01	-0.01	0.00	0.00	0.00	0.04	0.07	0.09	0.05
S7	0.03	0.00	-0.02	-0.02	-0.04	0.00	-0.14	-0.14	0.00
S8	0.00	0.01	0.01	0.03	0.05	0.06	0.13	0.12	0.05
S9	0.03	0.01	0.00	0.04	0.07	0.07	0.18	0.18	0.09
C	0.02	0.03	0.04	0.05	0.06	0.18	0.23	0.26	0

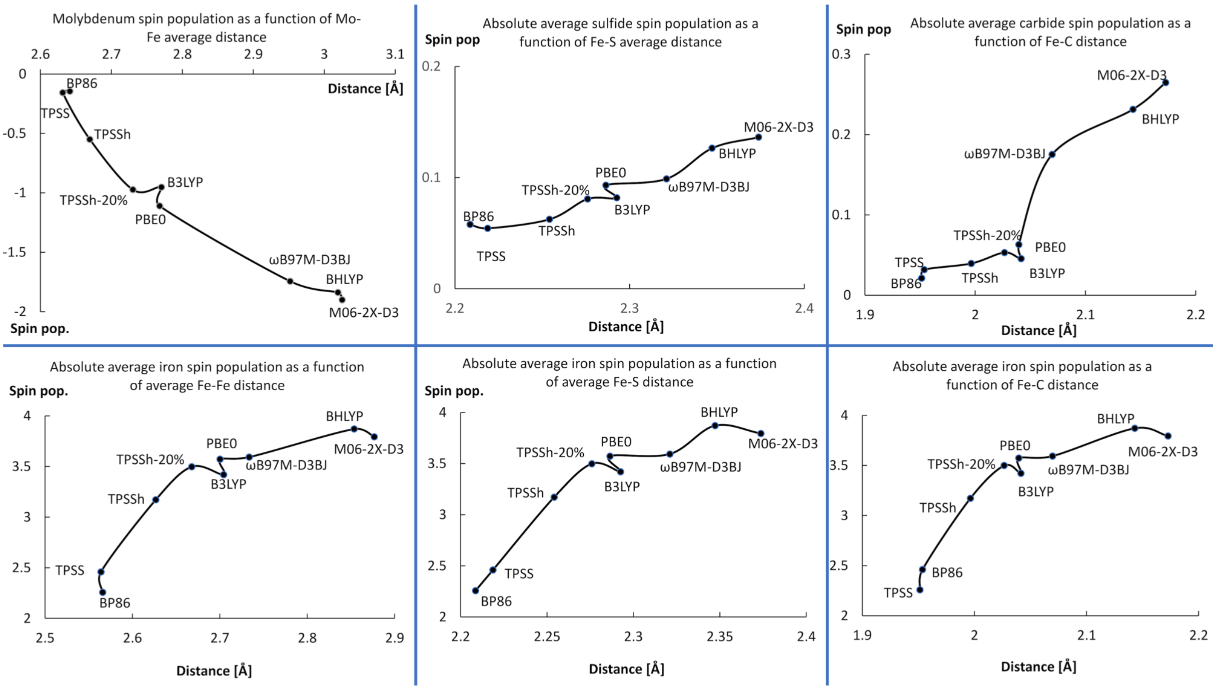

Figure S13. Atom spin populations (Mo,Fe,S,C) plotted as a function of various distances in the FeMoco cofactor for QM/MM calculations with different functionals.

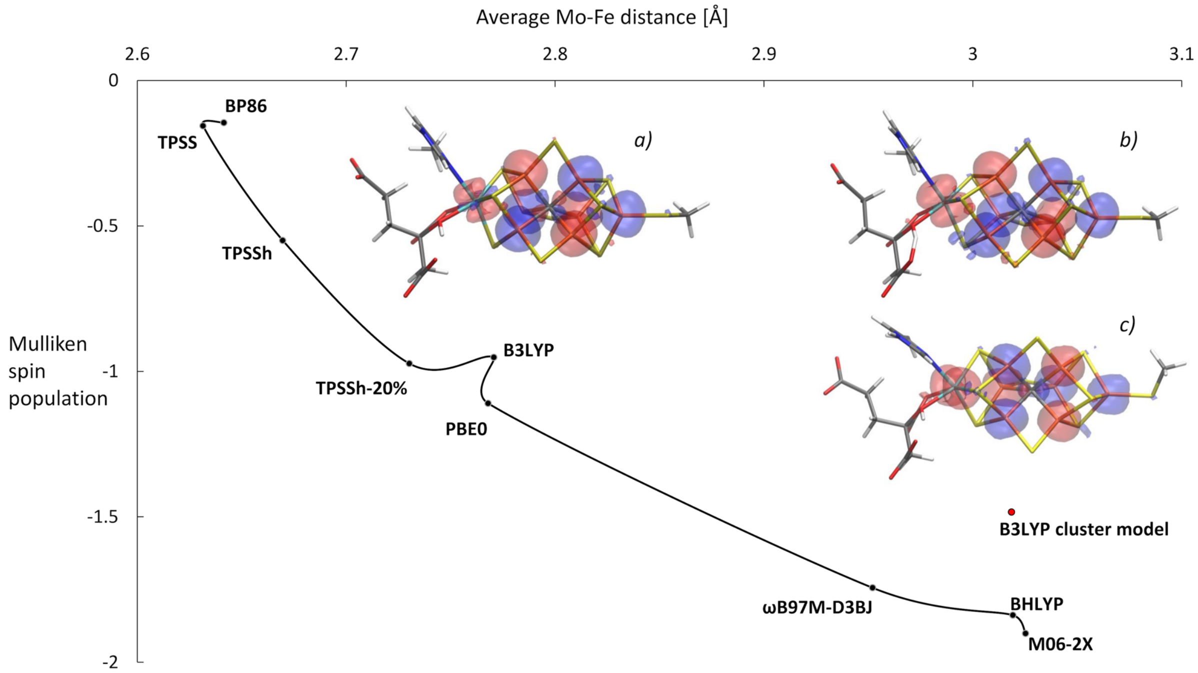

Figure S14. Mo spin population as a function of the average Mo-Fe distance for various QM/MM calculations with different functionals. Also shown are spin density isosurfaces with alpha spin colored in blue) and beta spin density colored in red. TPSSh calculation is labelled as *a)* , M06-2X calculation labelled as *b)*, and B3LYP cluster calculation labelled as *c)*. Note the lack of alpha spin density on Mo for surfaces in *b)* and *c)*.
